# Evaluation of a family-focused intervention to promote adolescent mental health and family relationships (phase 3 of MOST): protocol for a randomised controlled trial

**DOI:** 10.1136/bmjopen-2025-111877

**Published:** 2026-08-17

**Authors:** Janina Mueller, Swetha Sampathkumar, Dennis Wienand, Judit Simon, Antonio Piolanti, Marija Raleva, Viorel Babii, Ivo Kunovski, Nina Heinrichs, Franziska Waller, Bojan Shimbov, Rhiannon Evans, Graham Moore, Michael Radloff, Yulia Shenderovich, Heather M Foran

**Affiliations:** 1 Department of Health Psychology, University of Klagenfurt, Klagenfurt, Austria; 2 Centre for Development, Evaluation, Complexity and Implementation in Public Health Improvement (DECIPHer), School of Social Sciences, Cardiff University, Cardiff, UK; 3 Wolfson Centre for Young People’s Mental Health, Cardiff University, Cardiff, UK; 4 Department of Health Economics, Center for Public Health, Medical University of Vienna, Vienna, Austria; 5 Department of Psychiatry, University of Oxford, Warneford Hospital, Oxford, UK; 6 Institute for Marriage, Family and Systemic Practice – ALTERNATIVA, Skopje, Republic of North Macedonia; 7 University Clinic of Psychiatry, St Cyril and Methodius University, Skopje, Republic of North Macedonia; 8 Asociatia Obsteasca Sanatate Pentru Tineri (Health for Youth Association), Chisinau, Republic of Moldova; 9 Department of Psychology, Bielefeld University, Bielefeld, Germany; 10 Instituto de Economía Internacional, Department of Economics, University Jaume I Castellon, Castellón de la Plana, Spain

**Keywords:** Family, MENTAL HEALTH, Psychosocial Intervention, Randomized Controlled Trial, Adolescents

## Abstract

**Introduction:**

Parenting interventions have shown promise in improving mental well-being for parents and children and strengthening family relationships. Parenting for lifelong health (PLH) is an open-access, evidence-informed programme that aims to improve positive parenting practices, reduce family violence and promote mental health, particularly in low-resource settings. This study will evaluate the implementation and (cost-)effectiveness of an adapted version of the PLH for Parents and Teens programme. It will focus on supporting positive parenting, communication, mental health and well-being of adolescents and their caregivers in North Macedonia and Moldova.

**Methods and analysis:**

This multicountry hybrid type 1 effectiveness-implementation randomised waitlist-controlled trial will recruit 660 adolescents aged 10–14 years and their caregivers through schools, Youth Clinics and community partner organisations, including vulnerable and linguistically diverse families. The intervention group will receive the adapted PLH programme with assessments conducted postintervention and at 6-month follow-up. The waitlist control group will receive the adapted PLH programme after completion of the intervention group’s 6-month post-baseline assessment. Primary outcomes include adolescent emotional problems, family functioning, parenting practices, adolescent and caregiver quality of life. Secondary outcomes assess a range of mental well-being, family and other psychosocial outcomes. Other prespecified outcomes include implementation and cost outcomes. Primary analyses will compare intervention and waitlist control groups at postintervention and 6-month follow-up using intention-to-treat, baseline-adjusted linear mixed models. A within-trial economic evaluation will include cost–utility analysis and cost-effectiveness analysis. A macroeconomic analysis will assess broader economic impacts using public financing and budget impact analysis. The study uses a mixed-methods process evaluation, and integrates qualitative data, budget impact analysis and simulation modelling to inform scale-up considerations.

**Ethics and dissemination:**

The study has received ethical approval from all relevant sites. Results will be disseminated through peer-reviewed publications, scientific conferences and webinars, newsletters, social media and open-access platforms, alongside engagement with researchers, clinicians, policymakers and the public. The Family-Focused Adolescent & Lifelong Health Promotion project uses a targeted communication and dissemination strategy for families, implementers and policy stakeholders to promote the adoption and scale-up of evidence-informed, open-access parenting interventions for adolescents and caregivers in low-resource settings. Dissemination focuses primarily on North Macedonia and Moldova while also engaging actors across the wider Eastern European region.

**Trial registration number:**

NCT07240571.

**STRENGTHS AND LIMITATIONS OF THIS STUDY**

- This study uses the Multiphase Optimisation Strategy framework to systematically develop, refine and optimise the intervention through sequential preparation and factorial testing, integrating findings across all three phases (preparation, optimisation and evaluation) to strengthen confidence in the final intervention package.
- This study uses a type 1 hybrid effectiveness-implementation design with robust process evaluation and integrated economic evaluation to assess intervention effects, implementation, cost-effectiveness and scalability.
- This study uses targeted recruitment strategies to increase participation of linguistically diverse and socially vulnerable families, improving equity and inclusion.
- This study uses a 6-month follow-up period, which limits conclusions about the long-term sustainability of intervention effects.
- This study recruits families mainly through schools and service networks, which may limit generalisability to less service-connected populations.

## Introduction

 Adolescence is a transitional phase between childhood and adulthood and represents a critical period for the onset of mental health conditions and the formation of foundational health behaviours.^1 2^ Anxiety and depressive disorders are among the most common mental health conditions in youth.^3^ Positive relationships with family and peers can support adolescent health, whereas experiences such as bullying, social exclusion and conflict can contribute to emotional distress and mental health problems.^4^ Parental mental health and parenting styles can also have significant effects on adolescent health.^5^ Children exposed to family violence are at increased risk for a range of mental and physical health problems, and interparental conflict is associated with social anxiety and loneliness.^6^ In contrast, positive parenting practices and good communication are linked to better health outcomes.^7–9^ During adolescence, family relationships become particularly vulnerable, highlighting the importance of interventions that promote supportive parenting and reduce harsh parenting practices.^10^

Adolescents are also affected by broader social and environmental stressors. Poverty, housing instability and academic pressure can lead to chronic stress and mental health challenges.^11^ In Eastern Europe, youth face additional risks related to economic insecurity, post-COVID impacts, interpersonal violence and the ongoing war in Ukraine.^12^ In the Republic of North Macedonia and the Republic of Moldova, family separation due to migration or work abroad, as well as limited access to youth friendly mental health services, further increase vulnerability.^13 14^ The WHO recommends integrating mental health support into broader health strategies, including training for primary caregivers.^15^ Working with adolescents and caregivers on supportive relationships and good communication may help partly buffer the impacts of some of the wider risk factors.^16^

Programmes focusing on family relationships and parenting skills have emerged to be a promising strategy to enhance well-being and prevent mental health risks in children and young people.^17^ Such programmes aim to impart knowledge, embed skills and provide support to strengthen the caregiver and child relationships.^18^ Recent evaluation shows that early parenting interventions are effective in preventing child mental health problems across diverse cultures, clinical and socioeconomic settings.^19^ While originally programmes focused only on parents, increasingly they also involve older children and adolescents as active participants.

Parenting for lifelong health (PLH) is one such evidence-informed intervention, developed through collaboration among academic researchers, the WHO and UNICEF to support families in low- and middle-income countries.^20–23^ Randomised controlled trial (RCT) evidence showed that participation in the PLH for Young Children programme involving caregivers of children aged 2–9 years has been associated with significant improvements in positive parenting practices (Foran *et al*, forthcoming) and short-term reductions in child externalising behaviours, compared with an active control condition (Heinrichs *et al*, forthcoming) in Moldova, North Macedonia and Romania.

Although parenting interventions have shown promise in improving child and caregiver mental well-being, evidence on their impact on adolescent mental health remains limited.^24 25^ The current study will address this gap by evaluating the PLH for Parents and Teens programme, adapted with a specific focus on adolescent mental health, and using a range of relevant outcome measures. In addition, while interest in scaling up parenting programmes is growing, many studies focus primarily on effectiveness and pay less attention to implementation and scalability.^26 27^ To address this limitation, the study will include a process evaluation and consultations with stakeholders and families, including adolescents and caregivers, to inform programme adaptation and better understand opportunities for scale-up.

This study represents the final phase of the Family-Focused Adolescent & Lifelong Health Promotion (FLOURISH) project, which aims to (1) adapt, (2) optimise and (3) evaluate the implementation and (cost-)effectiveness of a PLH-based intervention targeting adolescents aged 10–14 and their caregivers in North Macedonia and Moldova (see Attachment 3 for more details on the other project phases). In this protocol, we describe plans for an RCT focusing on implementation, (cost)-effectiveness and scalability to address the following research questions:

1. How is the adapted PLH programme implemented by the intervention staff and how do the adolescents and caregivers participate in it? What is the variation in implementation and attendance?
2. What is the effectiveness of the adapted PLH programme on the primary outcomes postintervention and at 6-month follow-up?
3. What is the effectiveness of the adapted PLH programme on secondary outcomes postintervention and at 6-month follow-up?
4. What are the costs, quality of life outcomes and cost-effectiveness of the adapted PLH programme?
5. How do the stakeholders perceive the prospects for scaling-up the programme?
6. What are the required resources for scaling up the programme at national level and what is their budget impact?

This RCT will test the following hypotheses:

1. Adolescents and caregivers in the intervention group will show more favourable average scores on primary and secondary outcomes in the adapted PLH for Parents and Teens programme compared with the waitlist control group at post-intervention and at 6-month follow-up.
2. The adapted PLH programme will be more cost-effective compared with the waitlist control group, as measured by incremental cost per quality-adjusted life-year (QALY) and capability-weighted life year (CWLY) gained.
3. The adapted PLH programme will be implemented with high fidelity and participant attendance, with feedback from participants and staff indicating high satisfaction and positive perceived impacts.
4. Stakeholders will be supportive of programme scale-up and identify a range of opportunities and challenges related to the long-term funding of the programme.

## Methods

### Study design

The FLOURISH project is guided by the Multiphase Optimisation Strategy framework, implemented across three interrelated phases^28 29^: phase 1 (preparation),^30^ phase 2 (optimisation)^31^ and phase 3 (evaluation). This study (phase 3) uses a multicountry parallel-group RCT design with assessments at baseline, postintervention and 6-month follow-up to evaluate the adapted PLH programme for adolescents aged 10–14 years and their caregivers in North Macedonia and Moldova. This hybrid type 1 effectiveness-implementation trial examines implementation, (cost)-effectiveness and scalability.^32^ The intervention group will receive the adapted PLH programme immediately, while the waitlist control group will receive the programme after the intervention group’s 6-month follow-up.

The waitlist control design was selected to ensure that all participating families are eventually offered access to the intervention given the limited availability of comparable services in the participating settings. This approach was co-designed by researchers and implementers and was considered important for participant recruitment and retention. As part of the enrolment procedures, participants complete an exclusionary statement regarding acute distress and severe mental or physical health conditions that may interfere with participation. Individuals who self-identify as unable to participate due to health reasons are contacted by the research team and provided with information about appropriate support services. Enrolled participants are permitted to access usual care and other community-based support services during the trial. Participation in additional parenting programmes, counselling or mental health services will be recorded at each assessment to account for their potential influence on study outcomes.

To understand programme implementation and scalability, the trial includes an embedded mixed-methods process evaluation and public consultations. This approach aims to facilitate the adoption and dissemination of open-source, evidence-based family interventions for adolescents in low-resource settings.

The study was preregistered on ClinicalTrials.gov (NCT07240571; see Note), received ethical approval and will be reported in accordance with the Consolidated Standards of Reporting Trials (CONSORT) guidelines.^33^ The Standard Protocol Items: Recommendations for Interventional Trials (SPIRIT) Checklist for the protocol is also included as online supplemental appendix 1.^34^

### Study setting

Intervention implementation is conducted in two Eastern European countries, North Macedonia and Moldova, and is supported by two established health networks with prior experience delivering PLH programmes. In North Macedonia, the Institute for Marriage, Family and Systemic Practice (ALTERNATIVA) comprises a network of psychologists, social workers and family therapists engaged in research and education on systemic practice and family psychotherapy. In Moldova, the Health for Youth Association (HYA) supports publicly funded youth clinics that deliver preventive and therapeutic services addressing sexual and reproductive health, psychological well-being, substance misuse and violence prevention.

The intervention is scheduled to be delivered to the intervention group between fall 2025 and summer 2026. In Moldova, implementation will occur in a single wave, while in North Macedonia it will be conducted in two waves, based on the feasibility of recruiting families. This approach is consistent with procedures successfully used in North Macedonia during phase 2 of the FLOURISH project.

### Participants and recruitment

The study will include one primary adolescent and one primary caregiver per participating family. Secondary adolescents and secondary caregivers are also eligible to participate in the intervention, although their data will not be included in the primary analyses. Families will be recruited through the implementing organisations, ALTERNATIVA in North Macedonia and HYA in Moldova, as well as their affiliated service delivery networks. Recruitment will follow a structured process, with each group expected to include approximately 10 adolescent–caregiver pairs. Caregivers and adolescents aged 10–14 years will be first invited through multiple channels. After initial interest and verbal consent, country research coordinators will conduct screening interviews to determine eligibility. Table 1 summarises the eligibility and exclusion criteria for participating families.

**Table 1:** Inclusion and exclusion criteria for adolescents and caregivers

Participant	Inclusion criteria	Exclusion criteria
Adolescents	Aged 10–14 years at preassessment. Have lived with the caregiver in the same household for at least four nights per week over the past month. Provided informed assent. Adolescents aged ≥14 must provide informed consent in addition to caregiver consent (per North Macedonian legal/ethical guidelines). Fluent in at least one local language used in the study (Macedonian, Romanian, Russian or Albanian).	Severe psychological problems or acute mental disabilities (these participants will be referred to other mental health services). Participation in a previous phase of the FLOURISH project.
Caregivers	Aged ≥18 years at preassessment. Have lived with the adolescent in the same household for at least four nights per week over the past month. Provided written informed consent for themselves and the adolescent. Fluent in at least one local language used in the study (Macedonian, Romanian, Russian or Albanian).	Severe psychological problems or acute mental disabilities (these participants will be referred to other mental health services). Participation in a previous phase of the FLOURISH project.

FLOURISH, Family-Focused Adolescent & Lifelong Health Promotion; PLH, parenting for lifelong health.

Based on findings from previous phases of the FLOURISH project, recruitment in phase 3 will be expanded to better reach vulnerable families through both education and health system channels. In North Macedonia, this will include training school staff, psychologists, social workers and educational personnel to support recruitment efforts, as well as targeted outreach to families residing in family houses supported by SOS Children’s Village and Albanian-speaking communities. In Moldova, families will be recruited through Youth Klinic (YK) beneficiaries and health-promoting school partners using individual discussions, parent meetings at YKs and schools and informational announcements at YKs. Additional efforts will be made to include Russian-speaking families affected by displacement or regional instability related to the war in Ukraine and residents of the administrative-territorial units on the left bank of the Dniester river, also known as Transnistria.

Intervention staff will include facilitators, supervisors and supporting and coordinating personnel in each country, affiliated with the implementing organisations ALTERNATIVA in North Macedonia and HYA in Moldova. All intervention staff must be at least 18 years old, complete mandatory training, commit to their assigned roles in programme delivery, supervision or assessment, and provide written informed consent. An example of the caregiver informed consent form is provided in online supplemental appendix 2. Facilitators are professionals, some of whom have prior experience in parenting interventions, trained to deliver the PLH programme, lead group sessions and support adolescents and caregivers. Supervisors are senior staff members who oversee facilitators, monitor intervention fidelity and conduct facilitator supervisions and fidelity evaluations. Data assessors are trained research staff responsible for administering standardised assessments and ensuring high-quality, accurate data collection.

### Sample size

We performed a simulation-based power analysis for a baseline-adjusted linear mixed model with random intercepts for families nested in clusters, a fixed effect for treatment (PLH vs waitlist), timepoint (postintervention, follow-up), the treatment-by-time interaction and baseline measures as a covariate, using the R- package simr.^35^ We assumed an effect size of Cohen’s d=0.25 for the treatment effect at post-intervention, a within-person correlation of r=0.6 between baseline, post-intervention and follow-up measures, a between Intracluster Correlation Coefficient of 0.01, an alpha level of 0.05, and no further gains or losses at follow-up compared with post-intervention. Simulation results (1000 iterations) indicated that 62 clusters, each consisting of 10 families, achieve 84% power to detect an effect size of Cohen’s d=0.25 at postintervention and 82% power for the likelihood ratio test, comparing the model that includes the baseline covariate, time, the treatment main effect and the treatment-by-time interaction to the reduced model that contains only the baseline covariate and time. To achieve this sample size requirement in the presence of dropout, we plan to recruit 64 clusters and allow for cluster sizes slightly exceeding 10 families.

While the anticipated individual-level effects may be modest, as this is a preventive intervention, even small effects can translate into meaningful public health benefits when applied across a large number of individuals. Small shifts in risk or symptom trajectories, when sustained and broadly implemented, have the potential to prevent a substantial number of adverse outcomes and reduce downstream clinical burden.

### Randomisation and blinding

This study will involve a multicountry, cluster/group randomised controlled trial of the adapted PLH programme. Clusters will be randomly assigned to either the intervention group or the waitlist control group (see online supplemental appendix 3 figure S1 for the study flow chart). Each group (intervention and waitlist control) will consist of 10 adolescent–caregiver pairs, with a total of 32 groups per country, implemented across 16 sites in North Macedonia and 16 sites in Moldova.

Randomisation will occur at the group level and be based on predefined clusters with two clusters embedded within each geographical region (ie, school or youth clinic A and B). Stratified randomisation with country as the strata will be employed. A total of two sets with 16 sites/clusters will be randomised within each of the countries for a total of 64 sites/clusters overall. In North Macedonia, eligible site/clusters will be primary schools in Macedonian-speaking and Albanian-speaking urban and suburban areas of the Skopje metropolitan region. In Moldova, site/clusters will be Youth Clinics or schools. Across both countries, two clusters (ie, groups) per geographical region will be randomly assigned to either the intervention or the waitlist control condition.

The list of sites/clustered will be provided to the University of Klagenfurt, randomisation will be conducted independently by the University of Klagenfurt and provided to the research coordinators in North Macedonia and Moldova. The random allocation sequence will be generated by the project coordinator at the University of Klagenfurt using a computerised randomisation tool. Participants and data assessors will be blinded during informed consent and baseline assessments. After baseline is completed, participants will be informed of their allocation to intervention group or waitlist. Data assessors will remain blinded during the post and follow-up assessments. Pre, post and follow-up data sent to the analysis team will remain blinded until analyses are completed and shared with the team. To minimise assessment bias, different researchers will be responsible for outcome assessments and process monitoring.

### Intervention

A brief summary of the adaptation and optimisation of the PLH programme in phases 1 and 2 of the FLOURISH project is provided in online supplemental appendix 3 and described in Shenderovich *et al* and Piolanti *et al*.^30 31^ Below, we provide an overview of the version of the PLH programme that will be evaluated in phase 3.

Intervention group: Adapted PLH programme. The adapted PLH programme is designed to address key developmental and psychosocial needs by enhancing adolescents’ and caregivers’ communication, problem-solving and emotion regulation skills and strengthening family relationships, with the goal of preventing violence and improving positive parenting practices, ultimately benefiting mental well-being of adolescents and caregivers. It will be delivered over 7 weeks including a preprogramme visit and 6 weekly group sessions, each lasting approximately 2 hours, to groups of 10 adolescent–caregiver pairs. All sessions start and end jointly, and some sessions will also be completely conducted jointly, while parts of other sessions will be held separately for adolescents and caregivers to facilitate open discussion. Each group will be led by two facilitators who complete training on the PLH programme and receive ongoing supervision informed by video recordings to ensure fidelity. Supervisors also participate in trainings focused on supervision techniques and the use of the PLH Facilitator Assessment Tool (PLH-FAT).^36 37^ Session formats and content are detailed in table 2. Between-session home practice activities are included to support skill reinforcement. Programme materials for families and facilitators are freely available in English, Macedonian, Romanian, Russian and Ukrainian, and can be accessed through the FLOURISH website: https://www.flourish-study.org/study-materials.html. For participants who miss a group session, brief catch-up sessions are available.

**Table 2:** Adapted PLH programme

Week	Session	Adapted PLH programme for parents and teens	Content examples
1	0	Preprogramme visits	Introduction to the PLH programme for caregivers and teens
2	1	Developmental stages*	Introduction round Group rules House of support Biological, psychological and social changes during adolescence
3	2	Building a positive relationship through spending time together†	Quality time, trust and praise in family relationships
4	3	Talking about emotions and sensitive topics*	Dealing with positive and negative emotions I-statements Setting rules for speaking and listening
5	4	What do we do when we are angry*	Anger and stress management strategies Reframing thoughts
6	5	Establishing rules and routines†	Role play: importance of rules and routines Setting rules and routines and practice them
7	6	Problem solving*	5 steps to solve problems Saying no Celebration

* Separate session parts for adolescents and caregivers.

† Joint sessions with adolescents and caregivers.

To encourage participation, adolescents and caregivers receive a €5–€10 voucher (in local currency) after each assessment. In the final session, participants will also receive a certificate of attendance (for participation in fewer than five PLH sessions) or a certificate of successful completion (for attending five to six PLH sessions).

Control group: Waitlist control condition. The waitlist control group will receive the adapted PLH programme after the intervention group has completed the 6-month post-baseline assessment.

### Data collection and outcomes

Participants will be assessed at three time points: baseline, post-intervention (8–12 weeks after baseline) and at follow-up (6 months after baseline assessment) using computer-assisted self-administered questionnaires completed on tablets, computers or smartphones. Paper-based versions will be provided if necessary. Adolescents and caregivers will complete assessments separately, supported by trained data assessors. Assessors will receive training in data collection procedures, safety protocols and the monitoring and reporting of adverse events (AEs). All measures not previously available in the study languages were culturally adapted, translated and tested using a forward-translation and back-translation procedure conducted by bilingual mental health experts or professionals with mental health expertise (Piolanti *et al*, forthcoming). All measures and their assessment time points are listed in online supplemental appendix 3 tables S1–S3 and are briefly described below.

As a pragmatic evaluation of a complex intervention, the study includes five co-primary outcomes reflecting key domains of intervention impact. Outcomes such as family functioning and parenting practices are conceptualised as intermediary family processes expected to contribute to downstream adolescent mental health outcomes. Online supplemental appendix 3 figure S2 shows the conceptual model for the current study. Primary outcomes will be evaluated at post-intervention and 6-month follow-up to assess short-term and medium-term effects. Consistent with recommendations for trials of complex interventions, multiplicity will be addressed through prospective specification, transparent reporting and theory-driven interpretation of patterns of effects across the coprimary outcomes and time points.^38^

#### Primary outcomes

The study will assess primary outcomes reflecting key domains of adolescent mental health, family functioning, parenting practices and adolescent and caregiver health-related quality of life. Adolescent emotional problems (internalising) will be assessed using the caregiver-reported version of the Paediatric Symptom Checklist (PSC-17),^39^ internalising subscale. Family functioning will be measured using the caregiver-reported general functioning subscale of the Family Assessment Device (FAD-GF).^40^ Parenting practices will be assessed in caregiver report using the involved parenting and positive parenting subscales of the Alabama Parenting Questionnaire (APQ).^41^ For outcome assessment in the health economic evaluation, we will use information about health-related quality of life of adolescents using the EQ-5D-Y-3L and of caregivers using the EQ-5D-5L.^42^

#### Secondary outcomes

Adolescent emotional problems will be assessed using adolescent-reported symptoms on the Revised Child Anxiety and Depression Scale,^43^ including total, anxiety and depression scores, as well as adolescent-report and caregiver-report versions of the PSC-17,^39^ capturing internalising, externalising and attention problems. Family functioning related to problem solving will be measured using the caregiver-reported problem-solving subscale of the FAD.^40^ Family and parent—child communication will be assessed using the adolescent-reported Child-Parent Communication Apprehension Scale and the Parent–Child Communication Scale completed by both adolescents and caregivers.^44 45^ Additional secondary outcomes include parenting practices reported by adolescents and caregivers, assessed using the APQ, including involved and positive parenting and caregiver-reported harsh parenting.^41^ Caregiver stress will be assessed using the Parental Stress Scale.^46^ Social support will be measured for adolescents and caregivers using the emotional and affectionate support subscales of the Medical Outcomes Study Social Support Survey,^47^ while loneliness will be assessed using age-appropriate versions of the UCLA Loneliness Scale for adolescents Roberts UCLA Loneliness Scale (RULS-8) and caregivers RULS-6.^48 49^ Well-being will be measured for both adolescents and caregivers using the WHO-5 Well-Being Index (WHO-5).^50^ Caregiver psychological distress will be assessed using the Patient Health Questionnaire-9.^51^ Adolescent socioemotional skills, including emotional regulation, problem-solving and interpersonal skills, will be measured using the UNICEF Social Emotional Abilities and Learning tool.^52^ Exposure to emotional maltreatment will be assessed using the caregiver-reported emotional abuse subscale of the adapted ISPCAN Child Abuse Screening Tool, and adolescent experiences of peer violence and bullying will be measured using the Health Behaviour in School-aged Children Peer Violence measure (HBSC).^53–55^ Secondary outcomes related to the economic evaluation include the OxCAP-MH (Oxford CAPabilities questionnaire-Mental Health), an adult self-reported capability well-being instrument, which will be completed by caregivers.^56^ Further self-reported health and social care and broader resource use will be measured using the PECUNIA RUM (v2.0) self-assessed for caregivers and proxy assessed by caregivers for adolescents.^57^ All resources will be costed using national-level unit costs developed based on internationally validated unit costing methodology.^58^

#### Other prespecified outcomes

Other prespecified outcomes comprise measures of participant characteristics, intervention costs, implementation processes and staff outcomes that may influence programme effectiveness. These include food insecurity,^59^ adolescent healthy weight, adolescent and caregiver post-traumatic stress symptoms measured using the Children’s Revised Impact of Event Scale^60^ and the Posttraumatic Stress Disorder Checklist for DSM-5 (PCL-5),^61^ respectively, and adolescent top problems reported by adolescents and caregivers.^62^

Implementation outcomes will include participant and staff experiences. At baseline, staff will report demographic characteristics and prior professional experience. Potential intervention effects on staff will be assessed using pre–post measures of well-being (WHO-5)^50^ and parenting practices (APQ involved and positive parenting subscales).^41^ Staff will also complete weekly self-report measures on programme implementation and any programme modifications or adaptations made during delivery.

During the intervention period (6–8 weeks), implementation outcomes will include assessment of session fidelity using the PLH-FAT, monitoring of PLH session attendance (individual, family and catch-up attendance), and documentation of enrolment rates (individual and family enrolment).^58 63^ Weekly PLH sessions will be video-recorded for supervision and fidelity assessment purposes. A subset of sessions will be independently rated by supervisors using the 27-item PLH-FAT to generate fidelity scores for each intervention group. The fidelity assessment tool and coding guidance will be iteratively refined during rater training to support inter-rater reliability of at least 70% agreement and consistency of ratings across study sites.

Intervention cost data will be collected using centrally maintained consumable cost logs and weekly staff self-report time logs capturing training, delivery, supervision and administrative activities related to intervention implementation. These outcomes will be considered exploratory. Implementation outcomes will be assessed using quantitative and qualitative methods throughout intervention delivery and at postintervention assessment.

#### Process evaluation

To collect qualitative feedback on programme implementation, focus groups will be conducted after the intervention delivery with a sub-sample of adolescents, caregivers and intervention delivery staff from the intervention group. The focus groups will have around 6–8 participants with at least four focus groups conducted in each country (with caregivers, adolescents, facilitators and supervisors). In addition, approximately 20 family members from the intervention group families (eg, grandparents) who were not part of the dyad enrolled in the trial will be interviewed in each country to explore intervention experiences, acceptability and perceived impacts in the wider family. Furthermore, 10–15 interviews with professionals will be conducted per country to further progress understanding of the delivery context and potential scale-up (Shenderovich *et al*, forthcoming). A survey of the ALTERNATIVA and HYA networks will also be conducted to look at programme dissemination.

### Statistical analyses

Prior to effectiveness analyses, descriptive statistics, internal consistencies and correlations will be examined for all study variables. Descriptive statistics will be reported separately for each country and for the total sample. Drop-out analyses will be conducted to compare completers and non-completers of the assessments using analysis of variance and χ² tests. Primary analyses will compare intervention and waitlist control groups at post-intervention and 6-month follow-up using an intention-to-treat approach. No interim analyses are planned. Missing data will be handled via multiple imputation or full information maximum likelihood.

Treatment effects over time will be estimated using baseline-adjusted linear mixed models including fixed effects for treatment group, time and the treatment-by-time interaction, with random intercepts to account for families nested in groups and repeated observations. The treatment effect will be estimated using baseline-adjusted linear mixed models including treatment, time and treatment-by-time interaction terms. To this end, we will use linear mixed models with random intercepts, accounting for clustering at the group or site level, as appropriate, using robust standard errors or multilevel modelling approaches. Effect estimates, CIs and exact p values will be reported for the prespecified outcomes. Analyses will be performed in Mplus, Stata or R, using robust methods or multilevel models.

#### Health economic evaluation

We will conduct a within-trial economic evaluation, which will include a cost–utility analysis with cost per QALY and a cost-effectiveness analysis using cost per CWLY as respective outcome measures. The economic evaluation will be conducted from a health and social care sector perspective and will explore spill-over effects towards informal care, adolescent’s educational attainment and caregiver’s productivity losses. Costs of direct intervention delivery and costs due to caregiver self-reported and proxy-reported health and social care resource use will be considered. Our economic evaluation will yield incremental cost-effectiveness ratios and net benefit estimates comparing the intervention group to the wait-list group. Uncertainty will be assessed using bootstrapping and sensitivity analyses.

#### Quantitative implementation analyses

Quantitative process evaluation measures of programme implementation will be analysed using descriptive statistics, correlation and regression analyses. Associations of group session and catch-up session attendance with demographic characteristics of participants (eg, age, gender) will be checked for potential inequalities of access. Associations between facilitator experience and fidelity of delivery will be explored through correlation analyses.

Within the intervention group, associations will be explored between implementation indicators (eg, attendance and fidelity) and intervention outcomes. Indirect effect models will test the theories of change and implementation measures as mediators of outcome. Complier average causal effect models may also be employed to assess associations between intervention attendance and outcomes, depending on attendance distribution.

#### Qualitative analyses

The qualitative data collected from focus groups and interviews will be analysed using thematic analysis.

#### Scalability and financing strategies

We will gather stakeholder input, for example, through professional expert advisory groups, to assess the fiscal space and policy options for sustainable financing. We will conduct a macroeconomic analysis, including a public financing landscape summary, identifying funding mechanisms as well as a budget impact analysis based on coverage scenarios. We will produce a potential investment case for the intervention, including fiscal justification and prioritisation within the national budget. We plan to explore opportunities for scale-up and potential integration points within existing fiscal mechanisms in primary care, public education and local governments’ budget envelopes.

Furthermore, drawing on findings related to effectiveness, cost, cost-effectiveness and stakeholder views on scalability, simulation modelling will be used to examine potential scenarios for scaling the adapted PLH programme in Moldova and North Macedonia. We will conduct agent-based modelling, a simulation approach that models the outcomes of policy implementation based on three core components: agents, rules and environments.^42^ This method enables the investigation of complex, system-wide and long-term policy effects that are not feasible to evaluate through experimental designs. A scale-up strategy will be developed guided by this information.

#### Additional exploratory analyses

Additional exploratory analyses will compare the intervention and waitlist control groups on other prespecified outcomes at baseline, postintervention and 6-month follow-up. Multigroup analyses will be conducted to compare models across countries and demographic subgroups (eg, adolescent and caregiver gender). This analysis may inform and feed into the scale-up strategy in terms of potential coverage and outreach in the general population and inform the budget impact analysis of the intervention.

### Patient and public involvement

Advisory groups consultations with adolescents, caregivers, HYA and ALTERNATIVA staff and external professionals have been conducted in each phase of the FLOURISH project to obtain feedback on the results and to inform implementation and recruitment strategies, including for the randomised trial described here. Advisory group consultations will continue during the trial to inform programme scale-up considerations.

## Ethics and dissemination

The FLOURISH project has received ethical approval from (1) the Institutional Review Board for Research Ethics at the University of Klagenfurt in Austria (approval number: 2023-013), (2) the National Committee of Ethical Expertise for Clinical Trials of the Ministry of Health of the Republic of Moldova (approval number: 1476) and (3) the Ethical Commission for Human Research at the Medical Faculty, St. Cyril and Methodius University, Skopje, North Macedonia (approval number: 03-2144/4). Consistent with the Horizon Europe guidelines for clinical trials, modifications to the project are submitted in ethical amendments to the ethics boards.

The project adheres to established guidelines and recommendations to ensure clinical safety and efficacy. Oversight of ethics-related matters is provided by an independent Data Safety and Management Board (DSMB) and an Ethical Advisor (EA), who ensure compliance with key ethical standards, including the Declaration of Helsinki (2013). A Memorandum of Understanding between the project coordinator, the DSMB and the EA outlines the roles and responsibilities of each party. To safeguard both staff and participants, a Data Collection Safety Protocol and an AE Reporting Form were developed. All personnel involved in research activities and intervention delivery received training to identify and report AEs and serious AEs (SAEs), in accordance with the protocol and using the standardised AE form designed for the FLOURISH project. AE monitoring helps to detect any potential risks associated with assessments or intervention procedures. In the event of a SAE, a thorough investigation was carried out, and detailed reports were submitted to the relevant ethics committees and the DSMB to determine whether study procedures required modification. If it is decided that the SAE is related to the study, the DSMB and ethical boards may recommend changes. The DSMB and ethical boards will determine whether to proceed with the study, proceed with minor or major changes, or stop the study until concerns have been addressed. The feasibility and effectiveness of these safety procedures were assessed and updated during phase 1 to ensure robust monitoring throughout the project.

All data collected in FLOURISH will be handled with confidentiality and in compliance with the General Data Protection Regulation. A comprehensive data management plan was developed, outlining detailed procedures for data collection, access, deletion and secure storage. Prior to enrolment, participants are fully informed about the types of data to be collected. Data collected on paper will be stored in locked cabinets at the respective institutions responsible for data collection. Personal data (for participant contact and session monitoring during the intervention) and video recordings of the PLH sessions (for facilitator supervision and quality assessment) will not be shared across sites. This data will be securely stored locally in North Macedonia (ALTERNATIVA) and Moldova (HYA). Only select members of the local FLOURISH team will have access to this data. Anonymised data will be managed by the University of Klagenfurt, who is responsible for overall data management. Only anonymised datasets will be shared among project partners. To ensure data security, all project data will be backed up on two additional password-protected servers and/or external hard drives managed by the IT department at the University of Klagenfurt.

All project members have received training and certification in human subject research and ethical data management in accordance with relevant guidelines. The study protocol and statistical analysis plan will be made publicly available through the Zenodo repository and can also be obtained on reasonable request from the corresponding author. Anonymised quantitative data will be preserved and curated in Zenodo to ensure long-term accessibility.

The FLOURISH project uses a comprehensive communication and dissemination strategy tailored for families, implementers and other policy stakeholders. Dissemination efforts focus primarily on stakeholders in North Macedonia and Moldova to foster discussions on family policies, adolescent health and the scalability of parenting programmes, while also seeking to engage actors across the wider Eastern European region.

This multicountry hybrid type 1 effectiveness-implementation randomised waitlist-controlled trial aims to assess the implementation and (cost-)effectiveness of the adapted PLH programme in the Republic of North Macedonia and the Republic of Moldova. The study uses a waitlist control group as the comparator, allowing all participants to receive the adapted PLH programme while maintaining a rigorous evaluation of its effectiveness. Additionally, a scaling-up strategy will be developed, guided by a variety of data sources. This approach aims to advance the adoption and expansion of open-source, evidence-informed family interventions for adolescents and caregivers in low-resource settings, with the goal of reducing risks for mental health problems among families in Eastern Europe.

## Supplementary material

10.1136/bmjopen-2025-111877 online supplemental file 1

10.1136/bmjopen-2025-111877 online supplemental file 2

10.1136/bmjopen-2025-111877 online supplemental file 3

## References

[1] WHO Adolescent and young adult health. https://www.who.int/news-room/fact-sheets/detail/adolescents-health-risks-and-solutions.

[2] Steare T, Gutiérrez Muñoz C, Sullivan A (2023). The association between academic pressure and adolescent mental health problems: A systematic review. J Affect Disord.

[3] Racine N, McArthur BA, Cooke JE (2021). Global Prevalence of Depressive and Anxiety Symptoms in Children and Adolescents During COVID-19: A Meta-analysis. JAMA Pediatr.

[4] Lin J, Guo W (2024). The Research on Risk Factors for Adolescents’ Mental Health. Behav Sci (Basel).

[5] Anderson TL, Valiauga R, Tallo C (2025). Contributing Factors to the Rise in Adolescent Anxiety and Associated Mental Health Disorders: A Narrative Review of Current Literature. J Child Adolesc Psychiatr Nurs.

[6] Weymouth BB, Fosco GM, Mak HW (2019). Implications of interparental conflict for adolescents’ peer relationships: A longitudinal pathway through threat appraisals and social anxiety symptoms. Dev Psychol.

[7] Meeker EC, O’Connor BC, Kelly LM (2021). The impact of adverse childhood experiences on adolescent health risk indicators in a community sample. Psychol Trauma.

[8] Merrick MT, Ports KA, Ford DC (2017). Unpacking the impact of adverse childhood experiences on adult mental health. Child Abuse Negl.

[9] Strathearn L, Giannotti M, Mills R (2020). Long-term Cognitive, Psychological, and Health Outcomes Associated With Child Abuse and Neglect. Pediatrics.

[10] WHO WHO guidelines on parenting interventions to prevent maltreatment and enhance parent-child relationships with children aged 0–17 years. https://www.who.int/publications/i/item/9789240065505.

[11] Xu Z, Fu G (2025). The influencing factors and intervention pathways of adolescent emotional problems. *AJHSSR*.

[12] Ruokolainen H Mental health problems among young people are increasing – what can youth work do. https://www.oph.fi/en/news/2025/mental-health-problems-among-young-people-are-increasing-what-can-youth-work-do.

[13] Sandu V, Birtha M, Ianachevici M Situation analysis of children and adolescents in the Republic of Moldova. https://www.unicef.org/moldova/en/reports/situation-analysis-children-and-adolescents-republic-moldova.

[14] WHO WHO reignites action on mental health in North Macedonia. https://www.who.int/europe/news/item/16-05-2022-who-reignites-action-on-mental-health-in-north-macedonia.

[15] WHO Parenting for lifelong health: a suite of parenting programmes to prevent violence. https://www.who.int/teams/social-determinants-of-health/parenting-for-lifelong-health.

[16] Healy KL, Scott JG, Thomas HJ (2024). The Protective Role of Supportive Relationships in Mitigating Bullying Victimization and Psychological Distress in Adolescents. J Child Fam Stud.

[17] Kumpfer KL, Magalhães C (2018). Strengthening Families Program: An Evidence-Based Family Intervention for Parents of High-Risk Children and Adolescents. J Child Adolesc Subst Abuse.

[18] Han MX, Chesney E, Ng V (2025). Universal, selective and indicated parenting interventions to prevent the development of adverse mental health outcomes in youth: a meta-review of systematic reviews. *BMJ Ment Health*.

[19] Costantini I, López-López JA, Caldwell D (2023). Early parenting interventions to prevent internalising problems in children and adolescents: a global systematic review and network meta-analysis. *BMJ Ment Health*.

[20] Cluver LD, Meinck F, Steinert JI (2018). Parenting for Lifelong Health: a pragmatic cluster randomised controlled trial of a non-commercialised parenting programme for adolescents and their families in South Africa. BMJ Glob Health.

[21] Lachman JM, Heinrichs N, Jansen E (2019). Preventing child mental health problems through parenting interventions in Southeastern Europe (RISE): Protocol for a multi-country cluster randomized factorial study. Contemp Clin Trials.

[22] Shenderovich Y, Eisner M, Cluver L (2018). What Affects Attendance and Engagement in a Parenting Program in South Africa?. Prev Sci.

[23] Shenderovich Y, Eisner M, Cluver L (2019). Delivering a Parenting Program in South Africa: The Impact of Implementation on Outcomes. J Child Fam Stud.

[24] Backhaus S, Gardner F, Melendez-Torres GJ WHO guidelines on parenting interventions to prevent maltreatment and enhance parent–child relationships with children aged 0–17 years: report of the systematic reviews of evidence. https://cdn.who.int/media/docs/default-source/documents/violence-prevention/systematic_reviews-for-the-who-parenting-guideline-jan-27th-2023.pdf?sfvrsn=158fd424_3.

[25] Skeen S, Laurenzi CA, Gordon SL (2019). Adolescent Mental Health Program Components and Behavior Risk Reduction: A Meta-analysis. Pediatrics.

[26] Shenderovich Y, Lachman JM, Ward CL (2021). The Science of Scale for Violence Prevention: A New Agenda for Family Strengthening in Low- and Middle-Income Countries. Front Public Health.

[27] Peterson HB, Dube Q, Lawn JE (2023). Achieving justice in implementation: the Lancet Commission on Evidence-Based Implementation in Global Health. Lancet.

[28] Collins LM, Murphy SA, Strecher V (2007). The multiphase optimization strategy (MOST) and the sequential multiple assignment randomized trial (SMART): new methods for more potent eHealth interventions. Am J Prev Med.

[29] Collins LM, Nahum-Shani I, Guastaferro K (2024). Intervention Optimization: A Paradigm Shift and Its Potential Implications for Clinical Psychology. Annu Rev Clin Psychol.

[30] Shenderovich Y, Piolanti A, Babii V (2023). Family-focused intervention to promote adolescent mental health and well-being in Moldova and North Macedonia (FLOURISH): feasibility study protocol. BMJ Open.

[31] Piolanti A, Mueller J, Waller F (2025). Family-focused intervention programme to foster adolescent mental health and well-being: protocol for a multicountry cluster randomised factorial trial (FLOURISH Phase 2). BMJ Open.

[32] Curran GM, Bauer M, Mittman B (2012). Effectiveness-implementation hybrid designs: combining elements of clinical effectiveness and implementation research to enhance public health impact. Med Care.

[33] Schulz KF, Altman DG, Moher D (2010). CONSORT 2010 statement: updated guidelines for reporting parallel group randomised trials. BMJ.

[34] Chan A-W, Tetzlaff JM, Gøtzsche PC (2013). SPIRIT 2013 explanation and elaboration: guidance for protocols of clinical trials. BMJ.

[35] Green P, MacLeod CJ (2016). SIMR: an R package for power analysis of generalized linear mixed models by simulation. Methods Ecol Evol.

[36] Martin M, Lachman JM, Calderon F (2025). Scaling a parenting program to reduce child maltreatment in Tanzania: The role of facilitator fidelity in adolescent and parent outcomes. Child Prot Pract.

[37] Martin M, Lachman JM, Murphy H (2023). The development, reliability, and validity of the Facilitator Assessment Tool: An implementation fidelity measure used in Parenting for Lifelong Health for Young Children. Child Care Health Dev.

[38] Leroy JL, Frongillo EA, Kase BE (2022). Strengthening causal inference from randomised controlled trials of complex interventions. BMJ Glob Health.

[39] Murphy JM, Bergmann P, Chiang C (2016). The PSC-17: Subscale Scores, Reliability, and Factor Structure in a New National Sample. Pediatrics.

[40] Epstein NB, Baldwin LM, Bishop DS (1983). The McMaster family assessment device*. J Marital Family Therapy.

[41] Frick PJ (1991). Alabama Parenting Questionnaire (APQ) [database record]. APA PsycTests.

[42] Herdman M, Gudex C, Lloyd A (2011). Development and preliminary testing of the new five-level version of EQ-5D (EQ-5D-5L). Qual Life Res.

[43] Ebesutani C, Reise SP, Chorpita BF (2012). The Revised Child Anxiety and Depression Scale-Short Version: Scale reduction via exploratory bifactor modeling of the broad anxiety factor. Psychol Assess.

[44] Lucchetti A, Cursi M, Fiore D (2002). Development and validation of the child-parent communication apprehension scale. Interpers Commun Rev.

[45] Barnes HL, Olson DH (1982). Barnes and Olson communication scale [database record]. APA PsycTests.

[46] Berry JO, Jones WH (1995). Parental stress scale.

[47] Sherbourne CD, Stewart AL (1991). The MOS social support survey. Soc Sci Med.

[48] Roberts RE, Lewinsohn PM, Seeley JR (1993). A brief measure of loneliness suitable for use with adolescents. Psychol Rep.

[49] Wongpakaran N, Wongpakaran T, Pinyopornpanish M (2020). Development and validation of a 6-item Revised UCLA Loneliness Scale (RULS-6) using Rasch analysis. Br J Health Psychol.

[50] Topp CW, Østergaard SD, Søndergaard S (2015). The WHO-5 Well-Being Index: a systematic review of the literature. Psychother Psychosom.

[51] Kroenke K, Spitzer RL, Williams JB (2001). The PHQ-9: validity of a brief depression severity measure. J Gen Intern Med.

[52] UNICEF SEA baseline-endline tool. https://docs.google.com/forms/d/e/1FAIpQLSfsAm25WPFw0-oJip_P-LjVfwNhXMOo2C_cIjUIeqQqn6szGQ/viewform.

[53] Runyan DK, Dunne MP, Zolotor AJ (2009). The development and piloting of the ISPCAN Child Abuse Screening Tool-Parent version (ICAST-P). Child Abuse Negl.

[54] Jansen E, Frantz I, Hutchings J (2022). Preventing child mental health problems in southeastern Europe: Feasibility study (phase 1 of MOST framework). Fam Process.

[55] Cosma A, Molcho M, Pickett W (2024). A focus on adolescent peer violence and bullying in Europe, Central Asia and Canada. Health behaviour in school-aged children international report from the 2021/2022 survey. https://iris.who.int/handle/10665/376323.

[56] Simon J, Anand P, Gray A (2013). Operationalising the capability approach for outcome measurement in mental health research. Soc Sci Med.

[57] Pokhilenko I, Janssen LMM, Paulus ATG (2023). Development of an Instrument for the Assessment of Health-Related Multi-sectoral Resource Use in Europe: The PECUNIA RUM. Appl Health Econ Health Policy.

[58] Mayer S, Berger M, Perić N (2024). The Development of a New Approach for the Harmonized Multi-Sectoral and Multi-Country Cost Valuation of Services: The PECUNIA Reference Unit Cost (RUC) Templates. Appl Health Econ Health Policy.

[59] Cafiero C, Viviani S, Nord M (2018). Food security measurement in a global context: The food insecurity experience scale. Measurement.

[60] Perrin S, Meiser-Stedman R, Smith P (2005). The Children’s Revised Impact of Event Scale (CRIES): Validity as a Screening Instrument for PTSD. Behav Cogn Psychother.

[61] Price M, Szafranski DD, van Stolk-Cooke K (2016). Investigation of abbreviated 4 and 8 item versions of the PTSD Checklist 5. Psychiatry Res.

[62] Weisz JR, Chorpita BF, Frye A (2011). Youth top problems: Using idiographic, consumer-guided assessment to identify treatment needs and to track change during psychotherapy. J Consult Clin Psychol.

[63] Nagin DS, Sampson RJ (2019). The Real Gold Standard: Measuring Counterfactual Worlds That Matter Most to Social Science and Policy. Annu Rev Criminol.

