# Supplementary material for: Evaluation of a family-focused intervention to promote adolescent mental health and family relationships (phase 3 of MOST): protocol for a randomised controlled trial
Source: BMJ Open. 2026 Aug 17;16(8):e111877. doi: 10.1136/bmjopen-2025-111877 (PMC13536169; doi:10.1136/bmjopen-2025-111877)
Supplement: Supplementary file 1 [file bmjopen-2025-111877.reviewer_comments.pdf]

PEER REVIEW HISTORY

BMJ Open publishes all reviews undertaken for accepted manuscripts. Reviewers are asked to complete a checklist review form and are provided with free text boxes to elaborate on their assessment. These free text comments are reproduced below.

ARTICLE DETAILS

Title (Provisional)

Evaluation of a family-based intervention to promote adolescent mental health and family relationships (Phase 3 of MOST): Protocol for a randomized controlled trial

Authors

Mueller, Janina; Sampathkumar, Swetha; Wienand, Dennis; Simon, Judith; Piolanti, Antonio; Raleva, Marija; Babii, Viorel; Kunovski, Ivo; Heinrichs, Nina; Waller, Franziska; Shimbov, Bojan; Evans, Rhiannon E; Moore, Graham; Radloff, Michael; Shenderovich, Yulia; Foran, Heather M.

VERSION 1 - REVIEW

**Reviewer**	**1**
**Name**	**Chironda, Geldine**
**Affiliation**	**Seed Global Health**
**Date**	**30-Nov-2025**
**COI**	**None**

Evaluation of a family-based intervention to promote adolescent mental health and family relationships (Phase 3 of MOST): Protocol for a randomized controlled trial

Abstract

- Methods and Analysis: This protocol describes a multi-country randomized waitlist-controlled trial evaluating the implementation and (cost-)effectiveness of an adapted version of the PLH for Parents and Teens program in supporting positive parenting, enhancing communication, and improving mental health and well-being of adolescents and their caregivers in North Macedonia and Moldova. This is a very long sentence and the intended meaning is not clear. May you take out the aim of the study and make it standalone. In this section , only cover the design of the study.
- Methods and analysis: Overall, this section is too wordy, try to summarise further and most of the content can be unpacked in the methodology section.
- Methods and analysis: Statistical analyses will examine changes in outcomes over time, conduct a within-trial economic evaluation, and a macroeconomic analysis. Kindly indicate the exact statistical analyses to be used?

Introduction

- Randomized trial (RCT) evidence showed that participation in the PLH for Young Children program involving caregivers of children aged 2-9 years has been associated with significant improvements in positive parenting practices (Foran et al., forthcoming) and short-term reductions in child externalizing behaviors, compared to an active control condition (Heinrichs et al., forthcoming) in Moldova, North Macedonia, and Romania. Foran et al, Heinrichs et al, are they references for this content and if they are, they seem to be incomplete and should be referenced using the numbering system to match the rest of the document.
- In the present protocol, we report plans for an RCT of the Parenting for Lifelong Health (PLH) for Parents and Teens program. The Family-Focused Adolescent & Lifelong Health Promotion (FLOURISH) project aims to (1) adapt, (2) optimize, and (3) evaluate the implementation and (cost-)effectiveness of a PLH-based intervention package targeting adolescents aged 10-14 and their caregivers in North Macedonia and Moldova, with a focus on adolescent and caregiver mental health and well-being, and on program scalability. The intervention is designed to address key developmental and psychosocial needs by enhancing adolescents' and caregivers' communication, problem-solving, and emotion regulation skills. and strengthening family relationships, with the goal of preventing violence and improving positive parenting practices, ultimately benefiting mental well-being of adolescents and caregivers. This whole section is repeating some things. Kindly reframe and shorten it to clearly express the intended meanings.
- To achieve this objective, the project is guided by the Multiphase Optimization Strategy (MOST) framework, implemented across three interrelated phases.28, 29 Phase 1 (preparation) focused on adapting and pilot testing the intervention package, including the PLH for Parents and Teens program and three add-on components: UNICEF's Helping Adolescents Thrive tools (HAT), adolescent peer support, and engagement booster (Shenderovich et al., forthcoming; Mueller & Waller et al., forthcoming).30 In Phase 2 (optimization), a factorial trial was conducted to test different combinations of the adapted PLH program and three add-on components to identify the most effective and efficient configuration.31 The final Phase 3 (evaluation) will examine the impact of the intervention package, as identified in Phase 2, on adolescent (e.g., emotional problems) and caregiver (e.g., quality of life) outcomes, as well as family relationship outcomes (e.g., parenting practices). This whole section belongs to methodology, not in the introduction section.

Methodology

- There is need to reorganise the methodology section and reduce repetitions through out.
- Study setting: Intervention staff includes facilitators, supervisors, and support and coordinating staff in each country, affiliated with the implementing organizations – ALTERNATIVA in North Macedonia and HYA in Moldova. All intervention staff must be at least 18 years old, complete mandatory training, commit to their assigned roles in program delivery, supervision, or assessment, and provide written informed consent. Facilitators are professionals, some of whom have prior experience in parenting interventions, trained to deliver the PLH program, lead group sessions, and support adolescents and caregivers. Supervisors are senior staff members who oversee facilitators, monitor intervention fidelity, and conduct facilitator supervisions and fidelity evaluations. Data assessors are trained research staff responsible for

administering standardized assessments and ensuring high-quality, accurate data collection. This is for study population, not setting.

- Hypotheses of the study should be put just after the research questions, towards the end of the introduction
- To improve the flow of methodology, Sample size and randomisation and blinding should be covered just after population and recruitment

**Reviewer**	**2**
**Name**	**Kostyrka-Allchorne, Katarzyna**
**Affiliation**	**Queen Mary University of London, Psychology**
**Date**	**11-Apr-2026**
**COI**	**None**

Thank you for the opportunity to review the protocol for the study: "Evaluation of a family-based intervention to promote adolescent mental health and family relationships (Phase 3 of MOST): Protocol for a randomised controlled trial." The protocol concerns a multi-country RCT and implementation evaluation of a psychological group intervention for adolescents and their parents. This is a promising and relevant study, but important methodological details are currently unclear in the protocol. The key issues concern the description of outcomes, analysis, baseline assessment timing, and the implementation component. I hope my feedback summarised below will help the authors strengthen the protocol and improve the transparency of the study.

Major points

1. Clarity of outcomes

- Outcomes are mentioned only very broadly in the introduction, so it's not clear what the primary outcome is and which is the secondary outcome (as far as I can ascertain). It is difficult to determine whether the study is estimating change from baseline or the difference between groups at a follow-up time point (adjusted for baseline). The analysis section refers to both change scores and baseline-adjusted models, which creates confusion about the chosen analytical approach.

- In addition, the protocol appears to include multiple primary outcomes and possibly multiple endpoints, but this is not clearly stated or justified. The time point for the post-intervention assessment is also not clearly defined, and all time points should be explicitly anchored to randomisation.

- Overall, the outcomes need to be clearly defined, including what is being measured, at what time point, and how the treatment effect will be estimated. Key information about the outcomes should be included in the main manuscript rather than in the supplement.

2. Power calculation and analysis

- There is a discrepancy between the sample size calculation and the proposed analysis. The power calculation is based on ANOVA, whereas the analysis section refers to change models and ANCOVA. These approaches are not equivalent and have different assumptions and power implications.

- It is also not clear how the power calculation accounts for multiple outcomes or multiple endpoints. This makes it difficult to assess whether the study is sufficiently powered. The sample size justification should be aligned with the primary analysis, and the assumptions used should be clearly stated.

- Please clarify in the protocol whether interim analyses are planned and if any stopping rules have been agreed.

- Finally, is a small effect likely to have clinical importance? Please consider this and justify.

3. Timing of baseline assessment

- The protocol indicates that baseline assessment occurs after randomisation. This is a concern, as baseline measures are typically collected before allocation to avoid bias.

- If baseline data are collected after randomisation, participant responses may be influenced by their allocation or expectations, particularly for self-reported outcomes. The authors should clearly explain the sequence of randomisation and baseline assessment (currently, this is in the supplement and quite hard to find), justify this design choice, and describe how potential bias will be minimised.

4. Implementation component

- The implementation component is not described in sufficient detail. While the study is presented as a hybrid Type 2 design, it is not clear what the implementation outcomes are, how they will be measured, or at what time points.

- There is also a limited distinction between implementation outcomes and process evaluation. The research questions for the effectiveness and implementation components would benefit from being clearly separated. More detail is needed on the implementation outcomes, including their definitions, measurement methods, and planned analyses.

Additional points

- The abstract should state early on that this is a hybrid Type 2 effectiveness-implementation trial and briefly describe both components. It would also be helpful to specify where participants will be recruited from.

- The first two paragraphs of the introduction are quite dense and could be revised to focus on the most relevant background. Relatedly, some contextual information about earlier phases of the project that appears later in this section would be better placed earlier in the manuscript or moved to supplementary material.

- In the methods section, the study design should be clearly stated (for example, whether this is a parallel-group or a crossover trial), and the number and timing of follow-up assessments should be specified.

- The hypotheses should not appear in the middle of the method section. They are also very vague. For example, "Benefits of the adapted PLH program will outweigh relevant costs." Please specify what is meant by 'benefits' and 'costs', and does this refer to clinical or cost-effectiveness or implementation outcomes?

- The randomisation section includes lots of information about recruitment, which makes it a bit confusing. Please move this to the respective section and ensure that this section describes randomisation, allocation and blinding only, to improve clarity. Relatedly, this section mentions schools being randomised – if that is the case, this indicates a cluster randomised trial, which is a very different design to the one described in the other parts of the protocol.

- Please clearly separate the eligibility criteria for children and parents; perhaps it may be more effective to list the inclusion and exclusion criteria for each group in a small table.

- Please clarify where the families will be recruited from? Is it from the HYA and ALTERNATIVE organisations or from elsewhere?

- I appreciate the summary of the previous phases that involved the adaptation of the intervention, but this may be best moved to the supplement. The current study is already complex and includes multiple components, so it's best to reduce the amount of additional information summarised in the core document. It may be helpful to add some broad information about the content covered to provide some context for Table 1, though. Currently, the focus of the description is on the delivery.

- An explanation for the choice of comparator should be included in the protocol, and concomitant care that is permitted or prohibited during the trial also needs to be specified.

- Please clearly specify where the trial protocol and statistical analysis plan can be accessed – this information is currently not included in the protocol, as far as I can see.

- The strengths and limitations section does address some strengths, but not limitations.

VERSION 1 - AUTHOR RESPONSE

Reviewer: 1
Dr. Geldine Chironda, Seed Global Health
Comments to the Author:

Abstract

1. Methods and Analysis: "This protocol describes a multi-country randomized waitlist-controlled trial evaluating the implementation and (cost-)effectiveness of an adapted version of the PLH for Parents and Teens program in supporting positive parenting, enhancing communication, and improving mental health and well-being of adolescents

and their caregivers in North Macedonia and Moldova." This is a very long sentence and the intended meaning is not clear. May you take out the aim of the study and make it standalone. In this section , only cover the design of the study.

RESPONSE: Thank you for this helpful suggestion. For the abstract, we moved the study aim to the introduction section to present it as a standalone statement. In the methods and analysis section, we have revised the text to focus solely on the study design and shortened the sentence to improve clarity (page 3).

2. Methods and analysis: Overall, this section is too wordy, try to summarise further and most of the content can be unpacked in the methodology section.

   RESPONSE: We appreciate this feedback. We have further condensed the methods and analysis section of the abstract to improve clarity and readability (page 3).

3. Methods and analysis: "Statistical analyses will examine changes in outcomes over time, conduct a within-trial economic evaluation, and a macroeconomic analysis." Kindly indicate the exact statistical analyses to be used?

   RESPONSE: We have specified the statistical analyses to be used and added more detailed information to clarify the analytical approach (page 3).

Introduction

4. "Randomized trial (RCT) evidence showed that participation in the PLH for Young Children program involving caregivers of children aged 2-9 years has been associated with significant improvements in positive parenting practices (Foran et al., forthcoming) and short-term reductions in child externalizing behaviors, compared to an active control condition (Heinrichs et al., forthcoming) in Moldova, North Macedonia, and Romania." Foran et al, Heinrichs et al, are they references for this content and if they are, they seem to be incomplete and should be referenced using the numbering system to match the rest of the document.

   RESPONSE: We thank the reviewer for this comment. As the referenced material is not yet published, it cannot be included in the numbered reference list. In accordance with BMJ guidelines, this information has therefore been cited in the text only and not included as a formal reference (https://www.bmj.com/about-bmj/resources-authors/house-style).

5. "In the present protocol, we report plans for an RCT of the Parenting for Lifelong Health (PLH) for Parents and Teens program. The Family-Focused Adolescent & Lifelong Health Promotion (FLOURISH) project aims to (1) adapt, (2) optimize, and (3) evaluate the implementation and (cost-)effectiveness of a PLH-based intervention package targeting adolescents aged 10-14 and their caregivers in North Macedonia and Moldova, with a focus on adolescent and caregiver mental health and well-being, and on program scalability. The intervention is designed to address key developmental and psychosocial needs by enhancing adolescents' and caregivers' communication, problem-solving, and emotion regulation skills. and strengthening family relationships, with the goal of preventing violence and improving positive parenting practices, ultimately benefiting mental well-being of adolescents and caregivers." This whole section is repeating some things. Kindly reframe and shorten it to clearly express the intended meanings.

RESPONSE: That is a very valuable comment. We have reorganized the two paragraphs, shortened, and revised the section to remove repetition and improve clarity (page 5-6).

6. "To achieve this objective, the project is guided by the Multiphase Optimization Strategy (MOST) framework, implemented across three interrelated phases.28, 29 Phase 1 (preparation) focused on adapting and pilot testing the intervention package, including the PLH for Parents and Teens program and three add-on components: UNICEF's Helping Adolescents Thrive tools (HAT), adolescent peer support, and engagement booster (Shenderovich et al., forthcoming; Mueller & Waller et al., forthcoming).30 In Phase 2 (optimization), a factorial trial was conducted to test different combinations of the adapted PLH program and three add-on components to identify the most effective and efficient configuration.31 The final Phase 3 (evaluation) will examine the impact of the intervention package, as identified in Phase 2, on adolescent (e.g., emotional problems) and caregiver (e.g., quality of life) outcomes, as well as family relationship outcomes (e.g., parenting practices)." This whole section belongs to methodology, not in the introduction section.

   RESPONSE: The paragraph was moved to the Methods section (page 6). Information regarding the other phases of the project was moved to Appendix 2 of the Supplementary Materials.

Methodology

7. There is need to reorganise the methodology section and reduce repetitions through out.

   RESPONSE: We appreciate your advice and have reorganized the methodology section.

8. Study setting: "Intervention staff includes facilitators, supervisors, and support and coordinating staff in each country, affiliated with the implementing organizations – ALTERNATIVA in North Macedonia and HYA in Moldova. All intervention staff must be at least 18 years old, complete mandatory training, commit to their assigned roles in program delivery, supervision, or assessment, and provide written informed consent. Facilitators are professionals, some of whom have prior experience in parenting interventions, trained to deliver the PLH program, lead group sessions, and support adolescents and caregivers. Supervisors are senior staff members who oversee facilitators, monitor intervention fidelity, and conduct facilitator supervisions and fidelity evaluations. Data assessors are trained research staff responsible for administering standardized assessments and ensuring high-quality, accurate data collection." This is for study population, not setting.

   RESPONSE: Thank you for this suggestion. We have relocated the paragraph to the Participants and Recruitment section on page 7-8.

9. Hypotheses of the study should be put just after the research questions, towards the end of the introduction

   RESPONSE: Thank you for this helpful suggestion. We have revised the manuscript by moving the study hypotheses to immediately follow the research questions, as recommended, towards the end of the introduction (page 6).

10. To improve the flow of methodology, Sample size and randomisation and blinding should be covered just after population and recruitment

    RESPONSE: We have revised the structure of the Methods section by relocating the Sample Size section (page 8-9) and the Randomization and Blinding section (page 9) to follow immediately after the Participants and Recruitment section (pages 7-8).

Reviewer: 2
Dr. Katarzyna Kostyrka-Allchorne, Queen Mary University of London
Comments to the Author:

Thank you for the opportunity to review the protocol for the study: "Evaluation of a family-based intervention to promote adolescent mental health and family relationships (Phase 3 of MOST): Protocol for a randomised controlled trial." The protocol concerns a multi-country RCT and implementation evaluation of a psychological group intervention for adolescents and their parents. This is a promising and relevant study, but important methodological details are currently unclear in the protocol. The key issues concern the description of outcomes, analysis, baseline assessment timing, and the implementation component. I hope my feedback summarised below will help the authors strengthen the protocol and improve the transparency of the study.

<u>Major points</u>

11. Clarity of outcomes
    - Outcomes are mentioned only very broadly in the introduction, so it's not clear what the primary outcome is and which is the secondary outcome (as far as I can ascertain). It is difficult to determine whether the study is estimating change from baseline or the difference between groups at a follow-up time point (adjusted for baseline). The analysis section refers to both change scores and baseline-adjusted models, which creates confusion about the chosen analytical approach.
    - In addition, the protocol appears to include multiple primary outcomes and possibly multiple endpoints, but this is not clearly stated or justified. The time point for the post-intervention assessment is also not clearly defined, and all time points should be explicitly anchored to randomisation.
    - Overall, the outcomes need to be clearly defined, including what is being measured, at what time point, and how the treatment effect will be estimated. Key information about the outcomes should be included in the main manuscript rather than in the supplement.

      RESPONSE: We thank the reviewer for these important comments. We have revised the manuscript to improve clarity regarding the timing of assessments and the specification of study outcomes.

      First, we have summarized all primary, secondary, and other pre-specified in the manuscript under the data collection and outcomes section on page 12-13.

      Second, we have clarified the assessment time points for all measures in the manuscript (page 11) and in the supplementary document (Appendix 2: Tables S1-S3).

Third, we have clarified the use of multiple primary outcomes. The study includes five pre-specified co-primary outcomes reflecting key domains of the intervention's theoretical framework. Given that the intervention targets multiple interrelated domains of adolescent and family functioning, these outcomes were selected a priori to capture both direct and intermediary effects of the intervention. We have added a justification for this approach and clarified that outcomes will be assessed at both post-intervention and 6-month follow-up. In addition, we note that multiplicity will be addressed through prospective specification, transparent reporting, and theory-driven interpretation of effect patterns across outcomes and time points (page 12).

Finally, we thank the reviewer for pointing out the inconsistencies regarding the statistical analyses. We have now clarified the modelling approach in detail (page 13-14).

12. Power calculation and analysis
    - There is a discrepancy between the sample size calculation and the proposed analysis. The power calculation is based on ANOVA, whereas the analysis section refers to change models and ANCOVA. These approaches are not equivalent and have different assumptions and power implications.
    - It is also not clear how the power calculation accounts for multiple outcomes or multiple endpoints. This makes it difficult to assess whether the study is sufficiently powered. The sample size justification should be aligned with the primary analysis, and the assumptions used should be clearly stated.

    RESPONSE: Thank you for pointing out this important inconsistency. We have clarified the analytical strategy (page 13-14) and performed the corresponding power analysis on page 8-9 in the methods section under sample size. We do not plan to adjust alpha for the pre-specified primary outcomes as powering the study to test for small effects at lower alpha levels than 0.05 is not feasible given resource constraints.

13. Please clarify in the protocol whether interim analyses are planned and if any stopping rules have been agreed.

    RESPONSE: We thank the reviewer for this important comment. We confirm that no interim analyses are planned for this study, and this has now been explicitly stated in the revised manuscript (pages 13).

    Regarding stopping rules, no formal statistical stopping rules have been defined. However, a structured safety monitoring procedure is in place. All adverse events (AEs) and serious adverse events (SAEs) will be monitored across study sites and reported by Country Leads during scheduled assessments. SAEs will be reviewed by the Data and Safety Monitoring Board (DSMB) and the relevant ethics committees.

    If an SAE is determined to be related to the FLOURISH intervention, the DSMB and ethics committees may recommend appropriate actions, including continuation of the study, implementation of minor or major protocol modifications, or suspension/termination of the study until safety concerns are resolved. This has been clarified in the revised manuscript under the Ethics and Dissemination section (page 15).

14. Finally, is a small effect likely to have clinical importance? Please consider this and justify.

    RESPONSE: We have discussed this in the revised manuscript on page 9 in the Methods section under "Sample Size".

15. Timing of baseline assessment
    - The protocol indicates that baseline assessment occurs after randomisation. This is a concern, as baseline measures are typically collected before allocation to avoid bias.
    - If baseline data are collected after randomisation, participant responses may be influenced by their allocation or expectations, particularly for self-reported outcomes. The authors should clearly explain the sequence of randomisation and baseline assessment (currently, this is in the supplement and quite hard to find), justify this design choice, and describe how potential bias will be minimised.

    RESPONSE: Baseline assessments are conducted after cluster randomization. However, allocation is concealed at this stage. Randomization remains confidential, ensuring that both participants and data assessors are blinded to group allocation during the baseline assessment. As a result, the timing of baseline data collection is not expected to introduce bias. We have clarified this procedure in the manuscript to address this concern under the randomization and blinding section on page 9.

16. Implementation component
    - The implementation component is not described in sufficient detail. While the study is presented as a hybrid Type 2 design, it is not clear what the implementation outcomes are, how they will be measured, or at what time points.
    - There is also a limited distinction between implementation outcomes and process evaluation. The research questions for the effectiveness and implementation components would benefit from being clearly separated. More detail is needed on the implementation outcomes, including their definitions, measurement methods, and planned analyses.

    RESPONSE: We thank the reviewer for this helpful and constructive comment. We have revised the manuscript to improve clarity regarding the study design, the distinction between implementation outcomes and process evaluation, and the specification of implementation analyses.

    First, we have revised the study design classification from a hybrid Type 2 to a hybrid Type 1 effectiveness-implementation design (page 3, 4, 6, 16). This change reflects that the primary aim of the trial is to evaluate the effectiveness and cost-effectiveness of the adapted PLH intervention, while implementation is assessed as a secondary component. Importantly, the study does not include a separate or experimentally tested implementation strategy, which is a defining feature of hybrid Type 2 designs.

    Second, we have clarified and explicitly defined the implementation outcomes in the Methods section under "Data Collection and Outcomes" (Other Pre-specified Outcomes and Process Evaluation). The timing of these assessments is now explicitly stated in the manuscript (page 13), and further details are provided in the Supplementary Materials (Appendix 1).

Third, research questions 1 and 5 now specifically address the implementation components. These have been revised in the manuscript to address the reviewer's comment and to more clearly distinguish implementation from effectiveness objectives (page 6).

Fourth, additional details on the quantitative implementation analyses have been added (page 14).

Additional points

17. The abstract should state early on that this is a hybrid Type 2 effectiveness-implementation trial and briefly describe both components. It would also be helpful to specify where participants will be recruited from.

    RESPONSE: Thank you. As noted in our response to Comment 20, we have revised the study design. In addition, we have updated the abstract to clearly state the hybrid Type 1 effectiveness-implementation design. We have also added information specifying the participant recruitment setting and sources.

18. The first two paragraphs of the introduction are quite dense and could be revised to focus on the most relevant background. Relatedly, some contextual information about earlier phases of the project that appears later in this section would be better placed earlier in the manuscript or moved to supplementary material.

    RESPONSE: We thank the reviewer for this suggestion. The first two paragraphs of the introduction have been shortened to focus on the most relevant background. Information regarding the other phases of the project was moved to Appendix 2 of the Supplementary materials.

19. In the methods section, the study design should be clearly stated (for example, whether this is a parallel-group or a crossover trial), and the number and timing of follow-up assessments should be specified.

    RESPONSE: Thank you for this comment. We clarified in the methods section under study design that the study is a parallel-group randomized controlled trial with a waitlist control design, where groups are followed concurrently during the evaluation period and the control group receives the intervention after the 6-month follow-up assessment (page 6).

20. The hypotheses should not appear in the middle of the method section. They are also very vague. For example, "Benefits of the adapted PLH program will outweigh relevant costs." Please specify what is meant by 'benefits' and 'costs', and does this refer to clinical or cost-effectiveness or implementation outcomes?

    RESPONSE: We thank the reviewer for this important comment. We have revised the manuscript by moving the hypotheses to the Introduction section, immediately following the research questions, to improve clarity and structure. We have also revised the wording of the hypothesis to specify the outcomes more clearly (page 6).

21. The randomisation section includes lots of information about recruitment, which makes it a bit confusing. Please move this to the respective section and ensure that this section describes randomisation, allocation and blinding only, to improve clarity. Relatedly, this section mentions schools being randomised – if that is the case, this indicates a cluster randomised trial, which is a very different design to the one described in the other parts of the protocol.

    RESPONSE: We have revised the manuscript by moving all recruitment details to other sections, ensuring that the "Randomization and blinding" section now focuses exclusively on randomization procedures, allocation, and blinding (page 9).

22. Please clearly separate the eligibility criteria for children and parents; perhaps it may be more effective to list the inclusion and exclusion criteria for each group in a small table.

    RESPONSE: Thank you, we have clearly separated the eligibility criteria for adolescents and caregivers and presented the inclusion and exclusion criteria for each group in a concise table (Table 1) to improve clarity and readability (page 8).

23. Please clarify where the families will be recruited from? Is it from the HYA and ALTERNATIVE organisations or from elsewhere?

    RESPONSE: We have added clarification that families will be recruited through ALTERNATIVA in North Macedonia, HYA in Moldova, and their affiliated service delivery networks. This information has been included in the Methods section under "Participants and Recruitment" (page 7).

24. I appreciate the summary of the previous phases that involved the adaptation of the intervention, but this may be best moved to the supplement. The current study is already complex and includes multiple components, so it's best to reduce the amount of additional information summarised in the core document. It may be helpful to add some broad information about the content covered to provide some context for Table 1, though. Currently, the focus of the description is on the delivery.

    RESPONSE: The summary of the adaptation and optimization of the PLH program from Phases 1 and 2 has been moved to Appendix 2. More information about the content covered was added to Table 2 (page 11).

25. An explanation for the choice of comparator should be included in the protocol, and concomitant care that is permitted or prohibited during the trial also needs to be specified.

    RESPONSE: Thank you. We have clarified this in the Methods section under "Study Design" (page 6) and under "Ethics and Dissemination" (page 16).

26. Please clearly specify where the trial protocol and statistical analysis plan can be accessed – this information is currently not included in the protocol, as far as I can see.

    RESPONSE: The statistical analysis plan will be uploaded prior to commencement of any analyses. The following sentence was added under "Ethics and Dissemination" (page 16):

"The study protocol and statistical analysis plan will be made publicly available through the Zenodo repository and can also be obtained upon reasonable request from the corresponding author."

27. The strengths and limitations section does address some strengths, but not limitations.

    RESPONSE: We appreciate the reviewer's feedback. The section has been updated to also include study strengths and limitations (page 4).
