# Supplementary material for: Evaluation of a family-focused intervention to promote adolescent mental health and family relationships (phase 3 of MOST): protocol for a randomised controlled trial
Source: BMJ Open. 2026 Aug 17;16(8):e111877. doi: 10.1136/bmjopen-2025-111877 (PMC13536169; doi:10.1136/bmjopen-2025-111877)
Supplement: Supplementary file 3 [file bmjopen-16-8-s002.docx]

**Appendix 2: Informed Consent Phase 3**

License for the FLOURISH Consortium information sheet and consent form materials is held by the FLOURISH Consortium via Creative Commons Attribution NonCommercial 4.0 International Public License: https://creativecommons.org/licenses/by-nc/4.0/

**FLOURISH: Family-Focused Adolescent & Lifelong Health Promotion**

**Information sheet & consent form: Phase 3 (2025-2026) – Caregivers**

Thank you for your interest in this study. To participate, we need your written consent. Please read the following information carefully.

1. **What is the aim of the FLOURISH study?**

We want to find out whether a program works to support and improve adolescent wellbeing and family communication. The program focuses on caregiver-adolescent relationships and skills such as problem-solving and managing emotions, to reduce health risks for adolescents. We are inviting 720 families in Moldova and North Macedonia (360 in each country) to participate in this phase of the project.

2. **Why am I being asked to take part?**

We are asking parents or caregivers and their adolescents between the ages of 10-14 to participate in a study about families. By parent or caregiver, we mean any adult who is taking care of an adolescent – you do not have to be a biological parent or a legal guardian of the adolescent to participate. See section 5 for more details on who can take part in the study.

3. **What if I don’t understand something?**

This document may contain words or sentences that you do not understand. The research team can provide additional explanation. You can ask as many questions as you want to before deciding whether you want to take part. If you decide to take part, you can still ask as many questions as you want during or after the project.

4. **Do I have to take part?**

No. If you decide not to take part, you do not need to explain your reasons and there will be no consequences.

5. **Am I eligible to participate?**

We will see if you meet the necessary criteria and therefore ask you some questions.

- To participate in the study as a parent/caregiver you must be aged 18 or over.
- You must be the primary caregiver responsible for the care of an adolescent 10-14. You can be any caregiver – a biological or legal parent, or another family member.
- The adolescent must have lived with you at least four nights a week on average during the last month.
- The adolescent must also agree to take part in the program.
- You and your adolescent must be able to take part in a 6-week program (see Sec. 6, 14).
- You must be able to provide consent for both yourself and the adolescent (in the case that you are the legal guardian).
- You and your adolescent must agree to complete the surveys that ask questions about your thoughts, behaviors, and feelings.
- You, your adolescent, or another caregiver have not previously participated in any other phases of the FLOURISH project.

6. **Procedures of the study**

*Data collection*

All surveys will take place in school, community center, a university, or a similar location. You and your adolescent will be asked to choose a location that is convenient for you.

It is important for us to know how you and your adolescent are doing during and after the project. We will conduct the first survey before you participate in the group program. There will be separate surveys for you, and your adolescent. For each of you, the survey will take up to one hour. If you are currently in a relationship, we will also invite your partner to participate in the study, but they are not required to participate if they do not want to. If other adults or adolescents want to participate in the program, we will also invite them to complete the survey, but they are not required to do so if they do not want to. There will be support from the study team available to complete surveys. You do not have to answer any questions you don’t want to during these surveys and can leave any questions blank, without any consequence. Only adolescents and adults who complete the informed consent and first survey can participate in the program.

After the end of the program, we will contact you again to conduct the second survey with you and your adolescent. You will be asked similar questions to the questions at the beginning of the program. This will again take up to one hour. Finally, 6 months after the end of the program, we will contact you and your adolescent once again for a final survey, which will be similar to the previous two.

If you experience any difficulties, for instance feeling stressed, during the surveys or other research activities, or during the family program, please tell a staff member. We especially would like to know if you have any difficulties related to taking part in FLOURISH. The staff members will be trained and able to support you. (See also section 9).

*Family & adolescent program*

Before the program starts, you will meet in person or over the phone with the group leader who will give you more details about the program. Below, there is a brief overview of the main parts of the program. The program activities have been developed based on other programs that have been tested in research studies, recommended by international organisations, such as the World Health Organization and UNICEF, and with the input of caregivers, adolescents, and professionals in Moldova and North Macedonia.

**Parenting & family skills program, based on the “Parenting for Lifelong Health for Parents and Teens” program (6 group meetings)**

The program includes caregivers and adolescents and takes place over six 2-hour weekly sessions, delivered by two group leaders. During the group sessions, you and your adolescent will have activities on topics such as building a positive relationship and problem-solving and you will also practice at home. You will only do the activities if you want to. Some sessions will be conducted with adolescents and caregivers together, and some will be in separate groups of (1) adolescents and (2) caregivers. We will implement the program in community centres, schools, clinics, and similar places. Everything mentioned in the sessions will remain strictly confidential, unless someone is at immediate risk of harm (see section 10).

A person who is supervising the work of the group leaders (facilitators) will need to observe some of the group sessions to review the skills of the facilitator and give them advice. This is to help the facilitator build their skills in this new program. The observation of the session will not be looking at any individual caregivers or adolescents but focusing on the work of the facilitator. For this purpose, the sessions will be recorded on a video recorder, which will be set up to focus on the group facilitator. The video recording for quality assessment is a part of the program. Therefore, to take part in the study, all participants need to agree to the sessions being recorded.

*When will you receive the program?*

Everyone will receive the program, but at different times. To help us evaluate and improve the program, some families will receive the program soon after the first survey, and others will receive the program 6 months later. The time when you are invited to participate in the program will be determined by randomization (by chance).

7. **What happens if I change my mind and want to stop taking part?**

Participating in the study is voluntary. You can stop attending the program at any time and can stop taking part in the study at any time without consequences. You do not have to give a reason.

If you would like to stop taking part in the group sessions, you are free to stop attending them at any time. You can let your group facilitator know if you would not like to be contacted again about the family program. You would still be approached by the study team for a post-program survey, and participation in this is also voluntary.

If you want to leave the research study altogether, please contact: [in Moldova: Dr. Galina Lesco, xxxxxxxxxxxx, [xxxxxxxxxxxxxxxx](mailto:xxxxxxxxxxxxxxxx); in North Macedonia: Dr. Marija Raleva, +389 76456305, [marijaraleva@gmail.com](mailto:marijaraleva@gmail.com)] to withdraw from the study.

If you decide to leave the study, you can also request that your adolescent is withdrawn from the study too. If you would like to withdraw from the study yourself but you are still happy for your adolescent to participate, they can continue to participate if they want to, with your permission.

8. **Benefits and expense allowance**

You have the opportunity to participate in this caregiver-adolescent program for free. We don't know if participating in the program or certain group activities will help you personally, but we know that similar programs have helped other caregivers and adolescents around the world. The research findings will inform better service for families in your country and other countries. You and your adolescent will receive a certificate about your participation in the program, or a special certificate for high level of attendance. Additionally, you will both receive a 5-10 EUR (insert local currency) voucher for local purchases at each survey you complete.

**9. Risks and inconveniencies of the study**

In the survey, you and your adolescent will be asked questions about your personal experiences (e.g., thoughts, feelings) in various situations. Some questions may cause you or your adolescent certain feelings of sadness, anger or discomfort. If that happens, please let a member of the team know. Similarly, the group sessions could bring up something difficult, or it is possible that something you learn in the program could be difficult to do or cause an argument. If this happens, please notify the group leaders who will be able to support you. The team working on FLOURISH will be trained for these situations and informed about resources available for further support.

10. **Confidentiality of data**

Everything that you and your adolescent share with us as part of this study will remain confidential. The only exception may be potential harm to you or someone else. If we think you or someone else is at risk of being harmed, then members of the research team will consult with each other and decide what action to take based on country's Child Protection Standards and Policies.

Types of Personally Identifiable Information Collected:

**Contact Information and Code List**: We will ask for your contact information for scheduling purposes and when completing the consent forms. This information will be connected to a unique 4 number ID with a code list. The local research team will keep this code list of your unique four number ID and that of your adolescent in connection to your name and contact information. They will save this list in a password protected file on password protected computers and/or external hard drives in a locked room. Any paper copies of your contact information will be stored in a locked cabinet in the local research office and deleted as soon as entered in the computer. Only select members of the local research team will have access to this list.

**Video Recordings**: We would like to video-record program sessions as part of our quality survey and supervision of the program delivery. We will ask you and your adolescent to provide separate consent in this form for us to video-record sessions. The video material will be stored in a password protected file on password protected computers and/or external hard drives in a locked room at the HYA and IA centers. The video data will be used to rate the facilitator’s work and for discussion in supervision sessions for facilitators as part of their professional development, and not shared with anyone outside the country team and the IA/HYA networks. The country team may also use the videos for training of new staff in this program.

**Survey Responses during Sessions and throughout the Study**: The survey data collected in this study includes data on your background, wellbeing, and parenting experiences before and after the program, and 6 months after that, and attendance during the program. The survey data collected in the study for your adolescent includes questions related to their wellbeing and experiences before and after the program, 6 months after that, and attendance during the program. The survey answers you provide during the study will be labelled with the unique 4 number ID and stored separately from any personally identifiable information (your name, contact information, videos) in a password protected file on password protected computers and/or external hard drives in a locked room in the local team’s centers. Some questions may be asked in paper format and stored in a locked drawer in a locked room in the local team’s office. Only select members of the local research team will have access to this information. When your survey responses are shared with the international team, it will not include any way to identify how you are. Only select members of the local team will know your identity.

**Anonymized Data:** After the surveys are completed, your responses will be sent and stored electronically on the Open Data Kit software, in a password protected file on password protected computers and/or external hard drives at the University of Klagenfurt for at least 15 years after the completion of the study. You and your adolescent’s personal data will not be connected to any of your survey answers stored at the University of the Klagenfurt. This means the survey answers stored at the University of Klagenfurt will be anonymous, and will not identify you or any other research participant. Some anonymized data, such as anonymized survey responses, may be stored indefinitely for additional research.

**Data Storage and Deletion Procedures:** Your personally identifiable information will remain in your country, unless it is required for such an audit of the study. Only anonymized data will be shared with researchers in other countries. Within at most 15 years after the end of the study, the local research team in North Macedonia or Moldova will delete all personally identifiable information about you collected in connection with this research project. This means that the research team will delete your contact information and video materials or any information that identifies you or your adolescent. Your consent form will be retained for 15 years after the study completion and may be accessed by members of the research team and, where necessary, by members of the governance and audit teams of the  Partners conducting this research and by regulatory authorities.

We will publish information about the study results in academic journals, research reports and at conferences. Your name or other personal data will not appear there, and it will not be possible to identify you in any way. After the completion of the study, the research results will be published on the page: <https://www.flourish-study.org/about.html>.

11. **Can I withdraw my data?**

You or your adolescent may withdraw your permission to use your data and request that your survey responses are not used in analysis up until completion of the first survey. After you and your adolescent complete the first survey, it will be anonymously sent to the server in Klagenfurt and no longer possible to delete. You can also request to delete your survey data during subsequent surveys while those surveys are being completed, but not after. You may request that your or your adolescent’s personally identifiable information (e.g., from the consent forms, videos, or contact information) be deleted or your identity masked for as long as it is stored (i.e., up to 15 years after study completion) by contacting [in Moldova: Dr. Galina Lesco, xxxxxxxxxxxxxx, [xxxxxxxxxxxx](mailto:xxxxxxxxxxxx); in North Macedonia: Prof. Marija Raleva, +389 76456305, [marijaraleva@gmail.com](mailto:marijaraleva@gmail.com)].

12. **Review**

The Institutional Review Board for Research Ethics at the University of Klagenfurt evaluated this study and approved it on April 19^th^ 2023 (application 2023-013/1). Address: Universitätsstraße 65-67, 9020 Klagenfurt am Wörthersee, Austria E-Mail: [ethikrat@aau.at](mailto:ethikrat@aau.at). It has also been approved by the Ethics Board in Moldova on April 26th, 2023 (application 1476), National Committee of Ethical Expertise of clinical Trial of Ministry of Health of the Republic of Moldova, Chişinău; A. Cosmescu str, no.3, MD-2009, tel: +373 22 723000, email: [comitetetica@msmps.gov.md](mailto:comitetetica@msmps.gov.md) / North Macedonia on May 15th, 2023 (application number: 1978/1), Human Research Ethics Commission at the School of Medicine – St. Cyril and Methodius University Skopje, Macedonia, Str. 50 Divizija no. 6, 1000 Skopje.

13. **Who is responsible for this study?**

This study involves an international team of researchers and organizations at the University of Klagenfurt, Austria; Cardiff University, UK; Health for Youth Association, Republic of Moldova; Institute ALTERNATIVA, North Macedonia; University of Bielefeld, Germany; Association for Systematic Therapy Education Center, Serbia; Medical University of Vienna, Austria; and the University Jaume I Castellon, Spain.

Study website: <https://www.flourish-study.org/>

The main local contact person responsible for implementation of the study in Moldova is Dr. Galina Lesco and in North Macedonia is Prof. Marija Raleva. If there are any further questions in connection with this study, please contact the local study team: [in Moldova: Dr. Galina Lesco, xxxxxxxxxxxx, [xxxxxxxxxxxxx](mailto:xxxxxxxxxxxxx); in North Macedonia: Prof. Marija Raleva, +389 76456305, [marijaraleva@gmail.com](mailto:marijaraleva@gmail.com)]

Controller for Data Protection of the overall study at the University of Klagenfurt: [dsb@aau.at](mailto:dsb@aau.at)

14. **Exclusionary Statement**

This program is focused on teaching skills to improve well-being and communication for adolescents and their caregivers. This is not a substitute for psychiatric care, psychotherapy, or other medical services. If you are experiencing acute distress, suffering from an acute psychiatric disorder, or have any physical health conditions that may interfere with your participation, this program is not recommended for you at this time. We will contact you to follow up and provide referrals for other services that may be a better fit for your needs. Please select an option that fits best:

- 1. I am able to participate and am not experiencing acute distress or a mental health or physical health condition that would interfere with my participation.

OR

- 2. I am not able to participate due to health reasons.

*If you select option 2, the research team will follow up with referrals.*

15. **Declaration of consent and rights**

By signing and filling in your personal information, you confirm you agree with the following statements:

- I confirm that I have fully read and understood the study information sheet.
- Further questions were answered to my satisfaction by the study team.
- I understand that participating in the study is voluntary
- I understand that I can withdraw my participation or that of my adolescent’s during the study without giving reasons and without any disadvantages.
- I understand that what I say will remain confidential, unless I say something that indicates myself or someone else is at risk of harm/danger.
- I understand how my information and that of my adolescent’s will be stored, how it will be used, and that only the research team and funding regulators (for audit purposes) will have access.
- I understand I can revoke my consent to use of my personally identifiable information or that of my adolescent’s. This revocation does not impact the processing of data that was already carried out based on this consent until the point of revocation.
- With regard to my personally identifiable data or that of my adolescent’s stored during this study, I understand that I am generally entitled to the right to information, correction, restriction, transferability, objection and deletion, as well as the right to complain to the data protection authority.
- I understand I or my adolescent can quit at any stage of the program without any negative consequences.
- I understand that all sessions will be video recorded as part of quality assessment in the study.
- I can contact the office that oversees the research if I have questions about the study or about my rights (Moldova: National Committee of Ethical Expertise of clinical Trial of Ministry of Health of the Republic of Moldova, Chişinău; A. Cosmescu str, no.3, MD-2009, tel: +373 22 723000, email: [comitetetica@msmps.gov.md](mailto:comitetetica@msmps.gov.md); North Macedonia: Human Research Ethics Commission at the School of Medicine – St. Cyril and Methodius University Skopje, Macedonia; Str. 50 Divizija no. 6, 1000 Skopje).
- A copy of the informed consent document has been given to me.

**Consent for caregiver**

I hereby agree to participate in the study and consent with the processing of my personally identifiable information as part of this study.

Place: ______________________________________ Date: ______________________

First and last name of the participant: ___________________________________________

Signature of the participant: __________________________________________

**Consent for the participation of my adolescent (if legal guardian)**

I hereby agree for my adolescent to participate in the study and consent with the processing of my adolescent’s personally identifiable information as part of this study.

Place: _______________________________________ Date: ______________________

First and last name of the adolescent participant: ______________________________________

First and last name of the adolescent’s legal guardian: ________________________________

Signature of the adolescent’s legal guardian __________________________________________

**For Research Team**

Study team member to fill in both copies first:

First and last name of the study team member: ____________________________________

Signature of the study team member: _____________________________________

Funded by the European Union. Views and opinions expressed are however those of the author(s) only and do not necessarily reflect those of the European Union or UK Research and Innovation (UKRI). Neither the European Union nor the granting authorities can be held responsible for them. This work was funded by the European Union Horizon Europe research and innovation program under grant agreement number 101095528 and by the UKRI under the UK government’s Horizon Europe funding guarantee [grant number 10050850].

**FLOURISH: Family-Focused Adolescent & Lifelong Health Promotion**

**Information sheet & consent form: Phase 3 (2025-2026) - Adolescents**

|  | You are invited to participate in the FLOURISH study. |
| --- | --- |
| What is the study about? | We want to find out whether a program works to help adolescents feel good, and to help adolescents and caregivers communicate well and have good relationships. |
| Why have I been invited? | We are inviting adolescents aged 10-14 and their parents (or other adults, like grandparents, who take care of adolescents). 360 families in Moldova, 360 in North Macedonia will be invited to participate in this part of the project. |
|  | The group program for adolescents and caregivers will last for 6 weeks, with group meetings every week. The group will meet in a community center, clinic, or another similar place. |
|  | Everyone will receive the program, but at different times. To help us evaluate and improve the program, some families will receive the program soon, and others will receive the program 6 months later. |
|  | The group meetings will be led by one or two facilitators. Some weeks adolescents and adults will meet all together, and some weeks the meetings will be separate for adults and for adolescents. |
|  | A person who is giving advice on the work of the group facilitators (supervisor) will need to observe the group sessions. They will use video recordings of your group sessions to help guide the work of the facilitator. This is important for the supervisor to review the skills of the facilitator and give them advice. |
|  | It is important for us to know how you are doing during and after the project. We will conduct a survey (60 minutes) before the program starts, after it finishes, and 6 months after that. You will be asked to answer questions about your behaviors, feelings and relationships. |
| Why do you want my information? | We want to know whether this program works well and how it can be improved in the future. Honest answers are really important. |
|  | You and your caregiver will receive a (5-10 EUR, insert local currency) voucher at each survey you complete. |
| Do I have to take part? | No. It is your choice. If you don’t want to take part, you can tell us now or stop later at any time without any consequences. You do not have to tell us why. |
| Who will know what I say during the study? | Everything you tell us will be kept safe. We will not use your name when we tell or write to other people about the project. Everything you say in the group will be only used for research. One exception is if we are worried that you or someone else is in danger. Then we will have to tell someone about what you have told us, so they can help. |
| Who will know I participated? | You and your parent or another adult caregiver have to agree for you to participate. We will not tell anyone you participated in the study. |
|  | The program does not replace urgent medical services. If you have an urgent health problem that makes it impossible for you to participate, please let us know. We can give you a list of other services that may be more helpful for you right now. |
|  | We will keep your names and contact details, and the session videos for 15 years after the study and then delete them. |
| Who can I contact if I have more questions later? | You can contact the researchers who are there to answer any questions: Moldova: Dr. Galina Lesco, xxxxxxxxxxxxxx, [xxxxxxxxxxxxxxxxxx](mailto:galina.lesco@gmail.com)  North Macedonia: Prof. Marija Raleva, +389 76456305, [marijaraleva@gmail.com](mailto:marijaraleva@gmail.com)]  You can see more information about the project here: <https://www.flourish-study.org/about.html>. |
|  | **Did you understand everything? Do you have any questions?** |

**In the next section, we provide more detailed information about how your information is used and your rights as a participant in the study. We will explain this to you and to your caregiver in detail.**

**Detailed Description**

**Confidentiality of data**

Everything that you share with us as part of this study will remain confidential. The only exception may be potential harm to you or someone else. If we think you or someone else is at risk of being harmed, then members of the research team will consult with each other and decide what action to take based on country's Child Protection Standards and Policies.

Types of Personally Identifiable Information Collected:

**Contact Information and Code List**: We will ask for your contact information for scheduling purposes and when completing the consent forms. This information will be connected to a unique 4 number ID with a code list. The local research team will keep this code list of your unique four number ID in connection to your name and contact information. They will save this list in a password protected file on password protected computers and/or external hard drives in a locked room. Any paper copies of your contact information will be stored in a locked cabinet in the local research office and deleted as soon as entered in the computer. Only select members of the local research team will have access to this list.

**Video Recordings**: We would like to video-record program sessions as part of our quality survey and supervision of the program delivery. We will ask you to provide separate consent in this form for us to video-record sessions. The video material will be stored in a password protected file on password protected computers and/or external hard drives in a locked room at the HYA and IA centers. The video data will be used to rate the facilitator’s work and for discussion in supervision sessions for facilitators as part of their professional development, and not shared with anyone outside the country team and the IA/HYA networks. The country team may also use the videos for training of new staff in this program.

**Survey Responses during Sessions and throughout the Study**: The survey data collected in this study includes data on your background, wellbeing, and experiences before and after the program, and 6 months after that, and attendance during the program. The survey answers you provide during the study will be labelled with the unique 4 number ID and stored separately from any personally identifiable information (your name, contact information, videos) in a password protected file on password protected computers and/or external hard drives in a locked room in the local team’s centers. Some questions may be asked in paper format and stored in a locked drawer in a locked room in the local team’s office. Only select members of the local research team will have access to this information. When your survey responses are shared with the international team, it will not include any way to identify how you are. Only select members of the local team will know your identity.

**Anonymized Data**: After the surveys are completed, your responses will be sent and stored electronically on the Open Data Kit software, in a password protected file on password protected computers and/or external hard drives at the University of Klagenfurt for at least 15 years after the completion of the study. Your personal data will not be connected to any of your survey answers stored at the University of the Klagenfurt. This means the survey answers stored at the University of Klagenfurt will be anonymous, and will not identify you or any other research participant. Some anonymized data, such as anonymized survey responses, may be stored indefinitely for additional research.

**Data Storage and Deletion Procedures:** Your personally identifiable information will remain in your country, unless it is required for such an audit of the study. Only anonymized data will be shared with researchers in other countries. Within at most 15 years after the end of the study, the local research team in North Macedonia or Moldova will delete all personally identifiable information about you collected in connection with this research project. This means that the research team will delete your contact information and video materials or any information that identifies you. Your consent form will be retained for 15 years after the study completion and may be accessed by members of the research team and, where necessary, by members of the governance and audit teams of the Partners conducting this research and by regulatory authorities.

We will publish information about the study results in academic journals, research reports and at conferences. Your name or other personal data will not appear there, and it will not be possible to identify you in any way. After the completion of the study, the research results will be published on the page: <https://www.flourish-study.org/about.html>.

**Can I withdraw my data?**

You may withdraw your permission to use your data and request that your survey responses are not used in analysis up until completion of the first survey. After you complete the first survey, it will be anonymously sent to the server in Klagenfurt and no longer possible to delete. You can also request to delete your survey data during subsequent surveys while those surveys are being completed, but not after.

You may request that your personally identifiable information (e.g., from the consent forms, videos, or contact information) be deleted or your identity masked for as long as it is stored (i.e., up to 15 years after study completion) by contacting and requesting this in writing [in Moldova: Dr. Galina Lesco, xxxxxxxxxxxxxx, [xxxxxxxxxxxxxx](mailto:xxxxxxxxxxxxxx); in North Macedonia: Prof. Marija Raleva, +389 76456305, [marijaraleva@gmail.com](mailto:marijaraleva@gmail.com)].

**Who reviewed this study?**

The Institutional Review Board for Research Ethics at the University of Klagenfurt evaluated this study and approved it on April 19^th^ 2023 (application 2023-013/1). Address: Universitätsstraße 65-67, 9020 Klagenfurt am Wörthersee, Austria E-Mail: [ethikrat@aau.at](mailto:ethikrat@aau.at).

It has also been approved by the Ethics Board in Moldova on April 26th, 2023 (application 1476), National Committee of Ethical Expertise of clinical Trial of Ministry of Health of the Republic of Moldova, Chişinău; A. Cosmescu str, no.3, MD-2009, tel: +373 22 723000, email: [comitetetica@msmps.gov.md](mailto:comitetetica@msmps.gov.md) / North Macedonia on May 15th, 2023 (application number: 1978/1), Human Research Ethics Commission at the School of Medicine – St. Cyril and Methodius University Skopje, Macedonia, Str. 50 Divizija no. 6, 1000 Skopje.

**Who is responsible for this study?**

This study involves an international team of researchers and organizations at the University of Klagenfurt, Austria; Cardiff University, UK; Health for Youth Association, Republic of Moldova; Institute ALTERNATIVA, North Macedonia; University of Bielefeld, Germany; Association for Systematic Therapy Education Center, Serbia; Medical University of Vienna, Austria; and the University Jaume I Castellon, Spain.

None of your personally identifiable information will be shared with the international team. It will stay within your country (North Macedonia or the Republic of Moldova).

Study website: <https://www.flourish-study.org/>

The main local contact person responsible for implementation of the study in Moldova is Dr. Galina Lesco and in North Macedonia is Prof. Marija Raleva. If there are any further questions in connection with this study, please contact the local study team: [in Moldova: Dr. Galina Lesco, xxxxxxxxxxxxxxx, [xxxxxxxxxxxxxxxxx](mailto:galina.lesco@gmail.com); in North Macedonia: Prof. Marija Raleva, +389 76456305, [marijaraleva@gmail.com](mailto:marijaraleva@gmail.com)]

Controller for Data Protection of the overall study at the University of Klagenfurt: [dsb@aau.at](mailto:dsb@aau.at)

**Declaration of Consent for adolescent and rights**

- I confirm that I have fully read and understood the study information sheet.
- Further questions were answered to my satisfaction by the study team.
- I understand that participating in the study is voluntary
- I understand that I can withdraw my participation during the study without giving reasons and without any disadvantages.
- I understand that what I say will remain confidential, unless I say something that indicates myself or someone else is at risk of harm/danger.
- I understand how my information will be stored, how it will be used, and that only the research team and funding regulators (for audit purposes) have access.
- I understand I can revoke my consent to use of my personally identifiable information. This revocation does not impact the processing of data that was already carried out based on this consent until the point of revocation.
- With regard to my personally identifiable data stored during this study, I understand that I am generally entitled to the right to information, correction, restriction, transferability, objection and deletion, as well as the right to complain to the data protection authority.
- I understand I can quit at any stage of the program and nothing will happen.
- I understand that all sessions will be video recorded as part of quality assessment in the study.
- I can contact the office that oversees the research if I have questions about the study or about my rights (Moldova: National Committee of Ethical Expertise of clinical Trial of Ministry of Health of the Republic of Moldova, Chişinău; A. Cosmescu str, no.3, MD-2009, tel: +373 22 723000, email: [comitetetica@msmps.gov.md](mailto:comitetetica@msmps.gov.md); North Macedonia: Human Research Ethics Commission at the School of Medicine – St. Cyril and Methodius University Skopje, Macedonia; Str. 50 Divizija no. 6, 1000 Skopje).
- A copy of the informed consent has been given to me.
- My parent/caregiver has provided their consent for me to take part.

**Consent for adolescent**

I hereby agree to participate in the study.

Place: ______________________________________ Date: ______________________

First and last name of the participant: ___________________________________________

Signature of the participant: __________________________________________

**For Research Team**

Study team member to fill in both copies first:

First and last name of the study team member: ____________________________________

Signature of the study team member: _____________________________________

Funded by the European Union. Views and opinions expressed are however those of the author(s) only and do not necessarily reflect those of the European Union or UK Research and Innovation (UKRI). Neither the European Union nor the granting authorities can be held responsible for them. This work was funded by the European Union Horizon Europe research and innovation program under grant agreement number 101095528 and by the UKRI under the UK government’s Horizon Europe funding guarantee [grant number 10050850].
