# Supplementary material for: Evaluation of a family-focused intervention to promote adolescent mental health and family relationships (phase 3 of MOST): protocol for a randomised controlled trial
Source: BMJ Open. 2026 Aug 17;16(8):e111877. doi: 10.1136/bmjopen-2025-111877 (PMC13536169; doi:10.1136/bmjopen-2025-111877)
Supplement: Supplementary file 4 [file bmjopen-16-8-s003.docx]

**Appendix 3: Supplementary Materials**

**Adaptation and Optimization of the PLH Program in Phases 1 and 2 of the FLOURISH Project**

In the preparation phase (Phase 1), the intervention package – comprising the adapted PLH for Parents and Teens program and three additional components (adolescent mental health tools based on UNICEF’s Helping Adolescents Thrive (HAT), adolescent peer support, and an engagement booster) – was adapted to the contexts of North Macedonia and Moldova. The adaptation involved advisory group consultations with adolescents, caregivers, intervention staff, and professional experts, and context-mapping interviews. The intervention package was pilot-tested through a single-arm, multisite pre-post-trial. This phase also involved an evaluation of key implementation procedures, including recruitment, retention, and assessment strategies, and post-intervention focus group feedback from adolescents, caregivers, and intervention staff (Shenderovich et al., *forthcoming*). Findings from this pilot (N=64 adolescent-caregiver pairs) informed refinements to the intervention content, measurement instruments, and underlying conceptual model, thereby informing the design of subsequent project phases (Mueller & Waller et al., *forthcoming*).

In the optimization phase (Phase 2), a multi-country cluster randomized factorial trial was conducted to determine the optimal version of the intervention in terms of outcomes and cost consequences.^31^ Results from the factorial trial (N=653 adolescent-caregiver pairs) indicated that the adapted PLH program – excluding the three additional components (HAT, adolescent peer support, engagement booster) – was the optimal version of the intervention in terms of outcome and cost consequences. Based on these results, and the process evaluation findings (Sampathkumar et al., *forthcoming*), key adaptations for Phase 3 included the removal of the three add-on components from the intervention package, as well as revisions to the intervention content, informed consent procedures, and assessment measures. Intervention revisions included minor changes based on feedback from adolescents, caregivers, and intervention staff, such as replacing some physical exercises with energizers, and streamlining home practice discussions.

**Table S1.**

*Primary outcome measures*

| **Construct** | **Respondent** | **Measure** | **Assessment Time points** |
| --- | --- | --- | --- |
| Adolescent emotional problems  (internalizing) | Caregiver | Pediatric Symptom Checklist (PSC-17; Jellinek et al., 1986):^37^ internalizing subscale | Baseline, post-intervention (8–12 weeks after baseline), and 6-month follow-up |
| Family functioning  (general functioning) | Caregiver | Family Assessment Device (FAD-GF; Epstein et al., 1983):^38^ general functioning subscale | Baseline, post-intervention (8–12 weeks after baseline), and 6-month follow-up |
| Parenting practices (involved and positive parenting) | Caregivers | Alabama Parenting Questionnaire (APQ; Frick, 1991):^39^ involved parenting and positive parenting subscales | Baseline, post-intervention (8–12 weeks after baseline), and 6-month follow-up |
| Caregiver health-related quality of life | Caregiver | EQ-5D-5L (EuroQol Research Foundation, 2024)^40^ | Baseline, post-intervention (8–12 weeks after baseline), and 6-month follow-up |
| Adolescent health-related quality of life | Adolescents | EQ-5D-Y-3L (EuroQol Research Foundation, 2024)^40^ | Baseline, post-intervention (8–12 weeks after baseline), and 6-month follow-up |

**Table S2.**

*Secondary outcome measures*

| **Construct** | **Respondent** | **Measure** | **Assessment Time points** |
| --- | --- | --- | --- |
| Adolescent emotional problems | Adolescents | Revised Child Anxiety and Depression Scale (RCADS; Ebesutani et al., 2012):^41^ total score, depression and anxiety subscales | Baseline, post-intervention (8–12 weeks after baseline), and 6-month follow-up |
|  | Adolescents | Pediatric Symptom Checklist (PSC-17; Jellinek et al., 1986):^37^ total score, internalizing, externalizing and attention subscales | Baseline, post-intervention (8–12 weeks after baseline), and 6-month follow-up |
|  | Caregiver | Pediatric Symptom Checklist (PSC-17; Jellinek et al., 1986):^37^ total score, externalizing and attention subscales | Baseline, post-intervention (8–12 weeks after baseline), and 6-month follow-up |
| Family functioning (problem solving) | Caregiver | Family Assessment Device (FAD; Epstein et al., 1983):^38^ problem solving subscale | Baseline, post-intervention (8–12 weeks after baseline), and 6-month follow-up |
| Family communication | Adolescents | Child-Parent Communication Apprehension Scale (CPA-YA; Lucchetti et al., 2002)^42^ | Baseline, post-intervention (8–12 weeks after baseline), and 6-month follow-up |
| Parent-child communication | Adolescents & Caregiver | Parent-Child Communication Scale (Barnes & Olson, 1982)^43^ | Baseline, post-intervention (8–12 weeks after baseline), and 6-month follow-up |
| Parenting practices (involved and positive parenting) | Adolescents | Alabama Parenting Questionnaire (APQ; Frick, 1991):^39^ involved parenting and positive parenting subscales | Baseline, post-intervention (8–12 weeks after baseline), and 6-month follow-up |
| Parenting practices  (harsh parenting) | Caregiver | Alabama Parenting Questionnaire (APQ; Frick, 1991):^39^ corporal punishment subscale | Baseline, post-intervention (8–12 weeks after baseline), and 6-month follow-up |
| Parental stress | Caregiver | Parental Stress Scale (PSS; Berry & Jones, 1995)^44^ | Baseline, post-intervention (8–12 weeks after baseline), and 6-month follow-up |
| Social support  (emotional and affectionate support) | Adolescents & Caregiver | Medical Outcome Study Social Support Survey (MOS-SSS; Sherbourne & Stewart, 1991):^45^ emotional and affectionate support subscales | Baseline, post-intervention (8–12 weeks after baseline), and 6-month follow-up |
| Loneliness | Adolescents | UCLA Loneliness Scale (RULS-8; Roberts et al., 1993)^46^ | Baseline, post-intervention (8–12 weeks after baseline), and 6-month follow-up |
|  | Caregiver | Revised UCLA Loneliness Scale (RULS-6; Wongpakaran et al., 2020)^47^ | Baseline, post-intervention (8–12 weeks after baseline), and 6-month follow-up |
| Well-being | Adolescents & Caregiver | WHO-5 Well-Being Index (WHO-5; Topp et al., 2015)^48^ | Baseline, post-intervention (8–12 weeks after baseline), and 6-month follow-up |
| Psychological distress | Caregiver | Patient Health Questionnaire (PHQ-9; Kroenke et al., 2001)^49^ | Baseline, post-intervention (8–12 weeks after baseline), and 6-month follow-up |
| Socio-emotional skills | Adolescents | Social Emotional Abilities and Learning (SEAL) Tool (UNICEF, n.d.):^50^ emotional regulation, problem-solving and interpersonal skills subscales | Baseline, post-intervention (8–12 weeks after baseline), and 6-month follow-up |
| Emotional maltreatment | Caregiver | Adapted version of the ISPCAN Child Abuse Screening Tool-Intervention scale (ICAST; Runyan et al., 2009):^51^ emotional abuse subscale (5 items; Jansen et al., 2022)^52^ | Baseline, post-intervention (8–12 weeks after baseline), and 6-month follow-up |
| Peer violence and bullying | Adolescents | Health Behaviour in School-aged Children (HBSC) Study - Peer violence measure (4 items; Cosma et al., 2024)^53^ | Baseline and 6-month follow-up |
| Caregiver capability well-being | Caregiver | Oxford CAPabilities questionnaire - Mental Health (OxCAP-MH; Simon et al., 2013)^54^ | Baseline, post-intervention (8–12 weeks after baseline), and 6-month follow-up |
| Caregiver resource use | Caregiver | PECUNIA Resource Use Measurement (PECUNIA RUM; Pokhilenko et al., 2023)^55^ | Baseline, post-intervention (8–12 weeks after baseline), and 6-month follow-up |
| Adolescent resource use | Caregiver | PECUNIA Resource Use Measurement (PECUNIA RUM; Pokhilenko et al., 2023)^55^ – proxy version | Baseline, post-intervention (8–12 weeks after baseline), and 6-month follow-up |

**Table S3.**

*Other pre-specified outcome measures*

| **Construct** | **Respondent** | **Measure** | **Assessment Time points** |
| --- | --- | --- | --- |
| Food insecurity | Caregiver | Food Insecurity Experience Scale (FIES; Cafiero, Viviani & Nord, 2018)^56^ | Baseline, post-intervention (8–12 weeks after baseline), and 6-month follow-up |
| Adolescent healthy weight | Adolescents & Caregiver | Caregiver and adolescents report on the adolescents’ mass in kilograms and height in meters (BMI). | Baseline, post-intervention (8–12 weeks after baseline), and 6-month follow-up |
| Posttraumatic stress | Adolescents | Children’s Revised Impact of Event Scale (CRIES; Perrin et al., 2005):^57^ total score, intrusion and avoidance subscales | Baseline, post-intervention (8–12 weeks after baseline), and 6-month follow-up |
|  | Caregiver | PTSD Checklist for DSM-5 (PCL-5; Price et al., 2016)^58^ | Baseline, post-intervention (8–12 weeks after baseline), and 6-month follow-up |
| Adolescent top problems | Adolescents & Caregiver | Top Problem Assessment (TPA; Weisz et al., 2011)^59^ | Baseline, post-intervention (8–12 weeks after baseline), and 6-month follow-up |
| Well-being | Facilitators | WHO-5 Well-Being Index (WHO-5; Topp et al., 2015)^48^ | Baseline, post-intervention (8–12 weeks after baseline), and 6-month follow-up |
| Parenting practices (involved and positive parenting) | Facilitators | Alabama Parenting Questionnaire (APQ; Frick, 1991):^39^ involved parenting and positive parenting subscales | Baseline, post-intervention (8–12 weeks after baseline), and 6-month follow-up |
| Fidelity of intervention delivery | Facilitators | PLH-Facilitator Assessment Tool (PLH-FAT; Martin et al., 2023; Martin et al., 2025)^35,36^ | During intervention implementation (6–8 weeks) |
| Intervention costs | Intervention staff | Intervention costs will be calculated based on personnel time associated with the training and delivery of the intervention, organizational, material, transportation, and consumable expenses. | During intervention implementation (6–8 weeks) |
| PLH group session attendance  (individual attendance) | Intervention staff | Individual attendance for adolescents and caregivers will be calculated as the percentage of PLH group sessions attended (out of six). | During intervention implementation (6–8 weeks) |
| PLH group session attendance  (family and catch-up attendance) | Intervention staff | Family attendance will be assessed as the percentage of PLH sessions (out of six) attended jointly by the adolescent and caregiver. Catch-up session attendance will be assessed as the percentage of scheduled catch-up sessions attended (out of six). | During intervention implementation (6–8 weeks) |
| Enrollment rate  (individual and family enrollment) | Intervention staff | Percentage of enrolled adolescents and caregivers who attended at least one PLH session (individual enrollment) and percentage of families in which both attended at least one session (family enrollment). | During intervention implementation (6–8 weeks) |

**Figure S1.**

*Flow chart of multi-country randomized controlled trial (RCT)*

Recruitment of 660 adolescent-caregiver pairs (320-340 families per country)

Follow-up assessment

(6 month after baseline assessment)

Post assessment

(after intervention)

Intervention implementation

(1 pre-program visit + 6 PLH sessions)

Baseline assessment

Enrollment of 10 families per group

(caregiver + adolescent aged 10-14 years)

Randomization to:

- intervention group (adapted PLH core program)
- control group (waitlist control condition)

**Figure S2.**

*Conceptual model*

Communication^1,2^

Parenting Practices^1,2^

Parental Well-being and Stress^1^

Family Functioning^1^

Attendance^3^ Fidelity^3^

Core

Intervention

PLH

Teens

Internalizing Symptoms^1,2^

Implementation Outcomes

Adolescent Distal Outcomes

Family Outcomes

*Note*. ^1^ Reported by adolescents; ^2^ Reported by caregivers, ^3^ Reported by facilitators and supervisors
